# The Effects of Evidence Bounds on Decision-Making: Theoretical and Empirical Developments

**DOI:** 10.3389/fpsyg.2012.00263

**Published:** 2012-08-01

**Authors:** Jiaxiang Zhang

**Affiliations:** ^1^Cognition and Brain Sciences Unit, Medical Research CouncilCambridge, UK

**Keywords:** decision, boundary, integration, modeling

## Abstract

Converging findings from behavioral, neurophysiological, and neuroimaging studies suggest an integration-to-boundary mechanism governing decision formation and choice selection. This mechanism is supported by sequential sampling models of choice decisions, which can implement statistically optimal decision strategies for selecting between multiple alternative options on the basis of sensory evidence. This review focuses on recent developments in understanding the evidence boundary, an important component of decision-making raised by experimental findings and models. The article starts by reviewing the neurobiology of perceptual decisions and several influential sequential sampling models, in particular the drift-diffusion model, the Ornstein–Uhlenbeck model and the leaky-competing-accumulator model. In the second part, the article examines how the boundary may affect a model’s dynamics and performance and to what extent it may improve a model’s fits to experimental data. In the third part, the article examines recent findings that support the presence and site of boundaries in the brain. The article considers two questions: (1) whether the boundary is a spontaneous property of neural integrators, or is controlled by dedicated neural circuits; (2) if the boundary is variable, what could be the driving factors behind boundary changes? The review brings together studies using different experimental methods in seeking answers to these questions, highlights psychological and physiological factors that may be associated with the boundary and its changes, and further considers the evidence boundary as a generic mechanism to guide complex behavior.

## Neural Mechanisms of Perceptual Decisions

Making decisions on the basis of sensory information is a frequent and critical element of human lives. Imagine you are driving toward a traffic light in clear weather. You can easily decide to stop or accelerate depending on the color of the traffic light ahead. When driving in foggy weather, however, since the scene is less visible, it is more difficult to distinguish between the red and green light. You may need longer to make the correct decision, and may sometimes even make a mistake.

**Figure 1:**
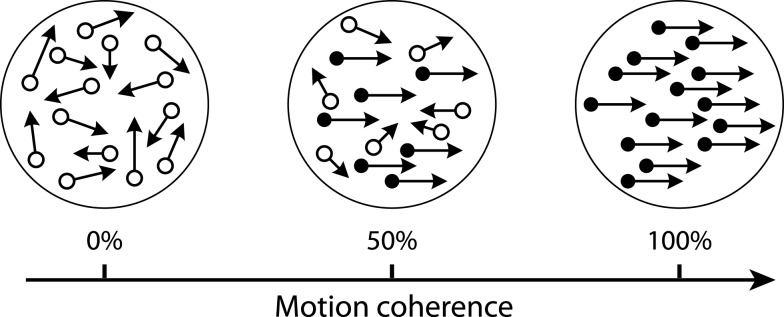
**Schematic diagram of the RDM stimulus with different motion coherence levels**. In each frame a proportion of the dots (solid dots) are repositioned with fixed spatial offset, indicating the coherent motion direction, and the rest of the dots (open dots) are repositioned randomly. More detailed specification of the stimulus is available in Britten et al. (1992).

This type of process is often referred to as perceptual decision-making (Newsome et al., 1989; Gold and Shadlen, 2001, 2007; Heekeren et al., 2008), which requires one to discriminate sensory attributes from either stationary or dynamic stimuli – such as an illumination with different colors (Yellott, 1971), a geometric shape with different orientations (Swensson, 1972), or a pixel array with different brightness (Ratcliff and Rouder, 1998) – and map the subjective perception onto multiple alternative responses. Laboratory studies of the decision process often employ one of two forced-choice paradigms. In the time-controlled (TC) paradigm, subjects are required to give their response immediately after a decision time set by the experimenter (Yellott, 1971; Swensson, 1972; Dosher, 1976, 1984). In the information-controlled (IC) paradigm, subjects are allowed to respond freely whenever they feel confident, from which subjects’ response times (RTs) can be measured as a second dependent variable (Luce, 1986). The neural mechanisms of perceptual decisions have been extensively studied using a prototypical random dot motion (RDM) discrimination task (Britten et al., 1993; Shadlen and Newsome, 2001; Roitman and Shadlen, 2002; Palmer et al., 2005; Churchland et al., 2008; Kiani et al., 2008). The RDM stimulus consists of a dynamic field of moving dots, a proportion of which move coherently in one direction, while the other dots move randomly (Figure 1). The task is to decide the direction of coherent motion and respond with an eye movement or a button press. Its difficulty can be manipulated by varying the strength of motion coherence.

Single-unit recordings in trained monkeys performing the RDM task indicate that the formation of perceptual decisions involve distinct neural processes across different brain regions. First, neuronal activity in motion sensitive areas (MT/V5; Maunsell and Van Essen, 1983; Born and Bradley, 2005; Zeki, 2007) are closely related to the statistics of the RDM stimulus (i.e., the motion coherence; Newsome and Pare, 1988; Salzman et al., 1990, 1992; Ditterich et al., 2003), but only weakly correlate with behavioral responses (Britten et al., 1992, 1993, 1996), suggesting that sensory neurons encode noisy, transient, and stimulus dependent evidence to support an alternative (Gold and Shadlen, 2001, 2007). Second, neurons in the lateral intraparietal (LIP) area respond with ramp-like changes, and the rate of change depends on the level of motion coherence (Shadlen and Newsome, 2001; Roitman and Shadlen, 2002). Unlike the MT neurons that respond transiently to visual stimuli, the LIP neurons gradually build up or attenuate their activity even if the visual stimuli remain ambiguous (i.e., 0% coherence). This activity pattern starts shortly after the stimulus onset and terminates before a saccadic response. Importantly, around ∼80 ms before a response, there is no obvious variability in firing rates of LIP neurons when responses are made under different motion coherence levels, and neural activity correlates only with the direction of eye movement (i.e., the decision). These findings suggest that LIP neurons integrate sensory evidence up to a decision boundary^1^ prior to a response (Mazurek et al., 2003; Huk and Shadlen, 2005; Hanks et al., 2006). Similar activity patterns have also been observed in other brain regions, including frontal eye fields (FEF; Schall, 2002), superior colliculus (SC; Basso and Wurtz, 1998), and dorsolateral prefrontal cortex (DLPFC; Kim and Shadlen, 1999). Taken together, these studies suggest a generic *integration-to-boundary* mechanism manifested in different brain regions for perceptual decisions. That is, certain neuronal populations integrate sensory information over time, and a response is committed to when the accumulated evidence reaches a decision boundary (Schall and Thompson, 1999; Gold and Shadlen, 2001, 2007; Heekeren et al., 2008).

The integration-to-boundary mechanism receives further support from psychological models of choice decisions that have been developed over the last half-century, namely sequential sampling models (Wald, 1947; Lehmann, 1959; Stone, 1960; Link, 1975; Link and Heath, 1975; Townsend and Ashby, 1983; Luce, 1986; Ratcliff and Smith, 2004; Smith and Ratcliff, 2004; Bogacz et al., 2006; Barnard, 2007). Sequential sampling models assume that evidence supporting alternatives are represented by a sequence of noisy observations over time. A process essential to reduce the noise in evidence is to integrate momentary observations over time and make a decision on the basis of the accumulated evidence. The sequential sampling models provide a detailed account of behavioral performance on choice tasks, including RT distributions, response accuracy, and relationships between the two (e.g., the speed–accuracy tradeoff). These models have been widely used as a mechanistic framework for isolating the decision process from sensory inputs or motor outputs.

A key prediction of almost all sequential sampling models is the presence of evidence boundaries, which limit the quantity of evidence available for making a decision. This article reviews recent theoretical and experimental developments in understanding the functions and mechanisms of the evidence boundary. The focus on the boundary mechanisms in general, rather than on particular decision models, is primarily due to its empirical relevance and importance. First, both experimental data and psychological models imply that the evidence boundary does not depend solely on sensory evidence, but can be internally set and controlled by a decision-maker. This unique characteristic of the boundary raises two important questions: (1) how can the evidence boundary influence decision performance? (2) How is the boundary implemented and adapted in neural circuits? Answers to such questions may provide insight into high-level cognitive control that subserves decision-making processes. Second, although the presence of the boundary is consistently supported by the neurophysiological (Mazurek et al., 2003; Huk and Shadlen, 2005; Hanks et al., 2006; Kiani et al., 2008) and neuroimaging (Ploran et al., 2007; Heekeren et al., 2008; Kayser et al., 2010a,b) data, only recently have researchers begun to investigate the function and effects of the evidence boundary. The understanding of its neural mechanisms is still insufficient.

The article is organized as follows: Section “Models of Decision-Making” reviews the decision-making problem and three representative sequential sampling models: the drift-diffusion model (DDM; Ratcliff, 1978), the Ornstein–Uhlenbeck (OU) model (Busemeyer and Townsend, 1993), and the leaky-competing-accumulator (LCA) model (Usher and McClelland, 2001). Section “Theoretical Considerations of Evidence Boundaries” examines the effects of the evidence boundary on the three models. This section discusses how the boundary may affect the models’ dynamics and fits to experimental data, and to what extent the boundary may affect the performance of these models. Section “Neural Implementation of Decision Boundary” and “Effects of Boundary Changes” review recent experimental findings that reveal possible neural underpinnings and behavioral influences on the decision boundary. Finally, Section “Discussion” offers some concluding remarks.

## Models of Decision-Making

### The decision problem and the optimal decision-making theories

Perceptual decision-making can be formalized as a problem of statistical inference (Gold and Shadlen, 2001, 2007). Let us consider a decision task with a choice between *N* (*N* ≥ 2) alternatives, each supported by a population of sensory neurons exclusively selective to a choice (e.g., motion sensitive neurons in area MT/V5). Stimuli drive the *N* populations of sensory neurons to generate noisy evidence streams *I_i_*(*t*) at time *t*, with mean μ*_i_* and variance $\sigma_{i}^{2}$ (*i* = 1, 2, 3, …, *N*). The goal of the decision process (e.g., reflected in activity of LIP neurons) is to identify which sensory population has the highest mean activity based on the evidence *I_i_*(*t*). This article mainly considers three representative models under this framework, as a more complete survey on sequential sampling models is available elsewhere (Ratcliff and Smith, 2004; Smith and Ratcliff, 2004; Bogacz et al., 2006).

Statistically optimal strategies exit for solving the decision problem with two alternatives (*N* = 2), which would achieve the lowest error rates (ER; the probability of making an incorrect choice in a block of trials) and the shortest RT compared with all other decision-making strategies. This optimality criterion can be divided into two sub-criteria (Bogacz et al., 2006): (1) the strategy yielding the lowest ER for any fixed amount of evidence, and (2) the strategy yielding the fastest response for any given ER. The two criteria correspond with the optimal conditions of the TC and IC paradigms, respectively. The optimal strategy for the TC paradigm, i.e., the lowest ER for fixed RT, is provided by the Neyman–Pearson test (NPT; Neyman and Pearson, 1933). The optimal strategy for the IC paradigm, i.e., the fastest RT for a given ER, is provided by the sequential probability ratio test (SPRT; Wald, 1947; Wald and Wolfowitz, 1948; Barnard, 2007). For multiple alternative decision tasks (*N* > 2), asymptotically optimal strategies are also available for the TC (Mcmillen and Holmes, 2006) and IC paradigms (Draglia et al., 1999; Dragalin et al., 2000).

Decision strategies that meet the optimal criteria above require linear integration of evidence over time, which, as reviewed below, can be implemented by many accumulator models on different level of abstraction (the implementation of optimal strategies for multiple alternative decisions requires models with additional complexity to those discussed here, see Bogacz and Gurney, 2007; Zhang and Bogacz, 2010b). Models that can accomplish optimal strategies have been shown to provide better explanations of experimental data than other, non-optimal, models (Ratcliff and Smith, 2004). This leads us to an ecologically motivated assumption that the brain may implement strategies for optimizing the speed and accuracy of decision-making, and hence optimal decision theories may offer a normative benchmark to generate experimental predictions and link behaviors to neural circuits for decision-making (Bogacz, 2007).

The perspective that the brain implements optimal decision-making relies on precise and circumspect definitions of the decision problem and criteria for optimality *per se*. For the simple decision problem with time-invariant evidence, linear integration is the optimal strategy in the sense of its speed and accuracy (see van Ravenzwaaij et al., 2012 for a discussion on other possible definitions of optimality). For tasks with time-varying signal-to-noise ratio within each trial (Huk and Shadlen, 2005; Tsetsos et al., 2011), linear integration may no longer be optimal. Intuitively, if the statistics and regularities of the time-varying evidence (i.e., when more reliable evidence arrives) are known, a decision strategy that exploits such knowledge and gives greater weight to more reliable evidence would have better performance than linear integration strategy (Papoulis, 1977). Whether humans are biased toward early or late evidence, or if their weights of evidence vary with practice (Brown and Heathcote, 2005b), or if their decision strategies are flexibly adapted (Brown et al., 2005), is still not fully understood and merits further investigation.

### Drift-diffusion model

**Figure 2:**
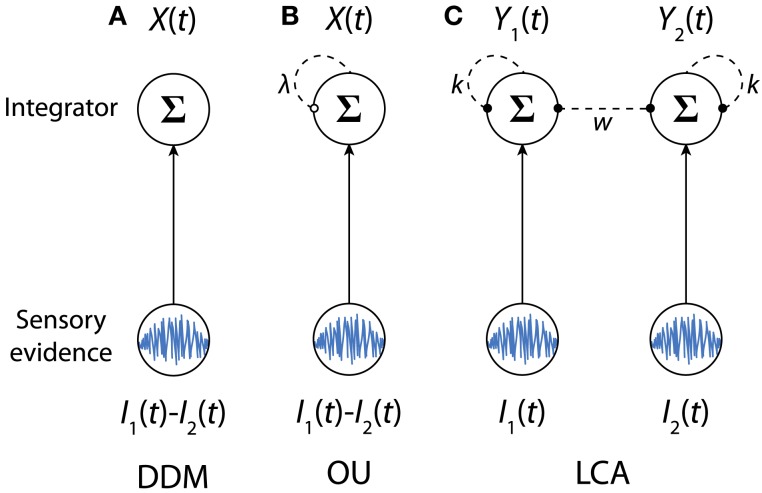
**The sequential sampling models for 2AFC tasks: (A) the DDM, (B) the OU model, (C) the LCA model**. Arrows denote excitatory connections. Dashed lines with solid circle end denote inhibitory connections. For the OU model, the dashed line with an open circle end denotes the effect of the growth-decay parameter. For each model, the bottom nodes denote sensory evidence, and the top notes denote neural integrators. Model parameters are defined in Eqs 1–3.

The DDM was proposed for two-alternative forced-choice (2AFC) tasks (Stone, 1960; Ratcliff, 1978). Mathematically, the DDM can be thought of as a standard Wiener process with external drift (Wiener, 1923), and is equivalent to a continuous limit of the random walk models (Estes, 1955; Laming, 1968; Link, 1975; Link and Heath, 1975; Luce, 1986). The model implies a single integrator that integrates the momentary difference between two sensory streams [*I*_1_(*t*) − *I*_2_(*t*)] supporting two alternatives (Figure 2A). The dynamics of the DDM can be characterized by a stochastic differential equation:

(1) $$dX\left(t\right)=\text{μ}dt+\text{σ}dW\left(t\right).$$

Here *dX*(*t*) denotes the increment of the accumulated evidence *X*(*t*) over a small unit of time *dt*. The sign of *dX*(*t*) implies that the momentary evidence at time *t* supports the first [*dX*(*t*) > 0] or the second [*dX*(*t*) < 0] alternative. μ is the drift rate of integration, representing the mean evidence difference (μ_1_ *–* μ_2_) per unit of time. If σ_1_ = σ_2_. The magnitude of μ is determined by the quality of the stimulus (the drift rate may be also determined by the allocation of attention, see Schmiedek et al., 2007). For example, for the RDM task, μ would represent the coherence level of the RDM stimulus: a large μ implies high motion coherence and an easy task, while a small μ implies low motion coherence and a high-level of difficulty in distinguishing between two coherent motion directions. The second term σ*dW*(*t*) denotes Gaussian noise with mean 0 and variance σ^2^*dt*. The DDM can be applied to either IC or TC paradigms. In the IC paradigm, decision time is unrestricted and two decision boundaries are introduced to indicate termination states (see Boundary Mechanisms). Once *X*(*t*) reaches a boundary, a corresponding choice is made. The predicted RT is equal to the duration of the integration, plus a non-decision time, corresponding to other cognitive processes unrelated to evidence integration (e.g., sensory encoding or response execution). For the TC paradigm, which requires subjects to respond at the experimenter-determined decision time *T_c_*, the model selects an alternative by locating the ultimate integrator state *X*(*T_c_*) and selecting the first alternative if *X*(*Tc*) > 0, or the second alternative if *X*(*T_c_*) < 0.

Several extensions of the DDM have been proposed since its original introduction, allowing model parameters to vary across trials. First, between-trial variability in the starting point of the integrator *X*(0) was introduced to account for premature sampling (Laming, 1968), which predicts faster errors than correct responses. Second, between-trial variability in the drift rate was introduced to account for slower errors when compared to correct responses (Ratcliff, 1978). The additional sources of parameter viabilities have been shown to improve fits to experimental data (Ratcliff et al., 1999).

The DDM have been applied to a number of cognitive tasks, including memory retrieval (Ratcliff, 1978), lexical decisions (Ratcliff et al., 2004a; Wagenmakers et al., 2008), letter identification (Ratcliff and Rouder, 2000), and visual discrimination including the brightness discrimination (Ratcliff, 2002; Ratcliff et al., 2003b) and the RDM task (Palmer et al., 2005). In all its applications, the model has successfully accounted for response accuracies and RT distributions observed from individual subjects (Ratcliff and Rouder, 1998; Ratcliff and Smith, 2004; Ratcliff and McKoon, 2008). More importantly, the simple DDM without between-trial parameter variability has been shown to implement the statistically optimal strategies for choosing between two alternatives (the NPT and the SPRT) in both TC and IC paradigms (Wald, 1947; Edwards, 1965; Gold and Shadlen, 2001, 2007; Bogacz et al., 2006), and hence the DDM is often used as a benchmark to compare the performance of other decision models. For the extended version of the DDM, previous studies suggest that the DDM with variable drift rate may still be the optimal model in the TC paradigm but the DDM with variable starting point is not optimal compared to other models (Bogacz et al., 2006). However a strict proof of the optimality of the DDM with between-trial visibilities is still not available yet.

One limitation of the DDM is that it was initially designed for binary choice tasks. Recent studies have attempted to extend the DDM to account for N-alternative forced-choice (NAFC) tasks (*N* > 2). One approach has been suggested by Niwa and Ditterich (2008). For a RDM task with three alternatives (i.e., three possible motion directions), Niwa and Ditterich (2008) modeled three integrators supporting the three alternatives rather than using a single integrator. The three integrators compete against each other in a race toward a common decision boundary and a response is determined by the winning integrator. Crucially, each integrator not only integrates sensory evidence supporting its preferred choice in a diffusion process, but also receives weighted feed-forward inhibition from evidence supporting the other two alternatives (Ditterich, 2010; see also Mazurek et al., 2003 for a similar approach). Churchland et al. (2008) proposed a slightly different approach for modeling a RDM task with four possible motion directions orthogonal to each other. Their hypothesis was that discriminating between two opposite motion directions (e.g., upper-left and lower-right) is independent of sensory evidence supporting the other two orthogonal directions (e.g., lower-left and upper-right). As a result, any sensory evidence supporting the two alternatives neighboring the true alternative was assumed to have a zero mean. The model nicely predicts a feature of their behavioral data that the probability for choosing the alternative directly opposing the true alternative is higher than that for the two alternatives neighboring the true alternative (Churchland et al., 2008). Leite and Ratcliff (2010) examined a family of models with multiple integrators in NAFC tasks with different number of alternatives (*N* = 2, 3, 4). Their results suggest that the models with independent integrators (i.e., no mutual inhibition) and zero to moderate decay produce qualitatively good fits to the RT distributions.

### Ornstein–uhlenbeck model

Similar to the DDM, the OU model has been proposed for 2AFC tasks (Busemeyer and Townsend, 1993), and has been applied to a variety of choice tasks to account for response accuracies and RT distributions (Heath, 1992; Diederich, 1995, 1997; Smith, 1995; Busemeyer, 2002). The OU model is identical to the DDM except that it includes a first-order filter that varies the change rate of an integrator (Busemeyer et al., 2006; Figure 2B). More precisely, the model is equivalent to a one-dimensional OU process (Uhlenbeck and Ornstein, 1930) and its dynamics can be described by the following differential equation:

(2) $$dX\left(t\right)=\left[\text{μ}+\text{λ}X\left(t\right)\right]dt+\text{σ}dW\left(t\right).$$

The drift rate μ and the noise term σ*dW*(*t*) have the same definitions as in Eq. 1 (see “Drift-diffusion model” above). The model contains a linear coefficient λ, a growth-decay parameter. As a result the rate of change of *X*(*t*) depends not only on the mean drift rate, but also on the current state of the integrator.

The growth-decay parameter brings some interesting properties to the OU model. First, in the TC paradigm, the response accuracy of the OU model reaches an asymptote for a large decision time *T_c_*. Note that the same prediction can be made from the DDM by introducing variability in drift rate across trials (Ratcliff et al., 1999), and that therefore theoretically the two models can account for behavioral data equally well (but, see Ratcliff and Smith, 2004). However, recent studies suggest that the two models are distinguishable by introducing temporal uncertainty to the stimulus (Huk and Shadlen, 2005; Kiani et al., 2008; Zhou et al., 2009). Second, the value of λ can account for the serial position effects observed in decision-making tasks (Wallsten and Barton, 1982; Busemeyer and Townsend, 1993; Usher and McClelland, 2001). For λ < 0, the linear term λ*X*(*t*) inhibits the integrator and the evolution of *X*(*t*) tends toward a stable attractor −μ*/*λ. Because evidence presented earlier in a trial decays over time, the choice mainly depends on the evidence later in the trial (a recency effect). In contrast, for λ > 0, the evolution of *X*(*t*) is repelled from the unstable fixed point −μ*/*λ, and the speed of repulsion is proportional to the distance between the current stage *X*(*t*) and −μ*/*λ. Therefore after *X*(*t*) has been driven to one side or other of the fixed point, subsequent evidence has little effect on the final choice due to repulsion (a primacy effect). For λ = 0, the OU model reduces to the DDM and hence implements the optimal decision strategy.

### Leaky-competing-accumulator model

The LCA model was proposed by Usher and McClelland (2001). Unlike the DDM and the OU model which integrate the relative evidence for one alternative compared with another, the LCA model assumes that evidence supporting different alternatives is integrated by separate integrators (Figure 2C). Therefore the LCA model can be naturally extended to account for decision tasks with multiple alternatives (Usher and McClelland, 2004; Mcmillen and Holmes, 2006; Tsetsos et al., 2011). Each integrator in the LCA model is leaky, as accumulated information continuously decays, and receives mutual inhibition from other integrators. For 2AFC tasks, the dynamics of the two integrators *Y*_1_(*t*) and *Y*_2_(*t*) can be described by:

(3) $$\left\{\;\begin{array}{c} dY_{1}\left(t\right)=\left({\text{μ}}_{1}-ky_{1}\left(t\right)-wy_{2}\left(t\right)\right)dt+\text{σ}dW_{1}\left(t\right)\\ dY_{2}\left(t\right)=\left({\text{μ}}_{2}-ky_{2}\left(t\right)-wy_{1}\left(t\right)\right)dt+\text{σ}dW_{2}\left(t\right) \end{array}\right.\;.$$

Here *k* (*k* ≥ 0) denotes the rate of decay, and *w* (*w* ≥ 0) denotes the weight of mutual inhibition from the other integrator. In the absence of sensory evidence (μ_1_ = μ_2_ = 0), the two integrators will converge to zero due to the effect of decay. The additional mutual inhibition means that the integrators are not independent, as each integrator can access the evidence that supports other alternatives. The LCA model can be applied to both IC and TC paradigms. In the IC paradigm, the first integrator that reaches a decision boundary renders its preferred choice. In the TC paradigm, the decision is determined by identifying which integrator has higher activity at a decision time *T_c_*. The model in Eq. 3 is a simplified linear version of the LCA model and the integrators’ values are unconstrained. In their original publication, Usher and McClelland (2001) assumed that the integrators’ stages are transformed by using a threshold-linear activation function, which prevents any integrator having negative values (Brown and Holmes, 2001; Brown et al., 2005). This non-linearity is motivated by the fact that activities of neural integrators can never be negative (see Boundary Mechanisms).

The LCA model is closely related to other sequential sampling models. For *w* = *k* = 0 (no decay or inhibition), the LCA model is equivalent to a model with independent integrators, which resembles a continuous version of the accumulator or counter models (Pike, 1966; Vickers, 1970). For 2AFC tasks, the LCA model can be reduced to an OU model if both decay and inhibition are large relative to the noise strength σ (Bogacz et al., 2006, 2007). The relative difference between *w* and *k* determines the growth-decay parameter λ in the reduced OU model (λ = *w* − *k*). That is, if the inhibition is larger than the decay (*w* > *k*), the LCA model can be reduced to an OU model with λ > 0. In contrast, if the inhibition is smaller than the decay (*w* < *k*), the LCA model can be reduced to the OU model with λ < 0. Therefore, similar to the OU model with λ ≠ 0, the LCA model with unbalanced inhibition and decay (*w* ≠ *k*) can account for primacy and recency effects (Usher and McClelland, 2001). For balanced decay and inhibition (*w* = *k*), the LCA model can be approximated by the DDM and hence implements the optimal decision strategy.

Because the LCA model can mimic the DDM and the OU model within a certain parameter range, the LCA model retains the strength of the simpler models to account for detailed aspects of behavioral data from 2AFC tasks. The LCA model has also been successfully applied to perceptual decision tasks with multiple alternatives (Usher and McClelland, 2001; Tsetsos et al., 2011), and value-based decisions, in which the decisions are settled on subjective preferences, rather than perceptual information (Usher and McClelland, 2004; Usher et al., 2008).

### Decision-making models at different levels of complexity

The sequential sampling models do an excellent job of accounting for the variability of responses and RTs in various decision tasks. Over decades researchers have tended to extend existing models to account for more systematic effects (e.g., RT differences between correct and error responses) or more biologically realistic constraints (e.g., the mutual inhibition and decay in the LCA model). These attempts led to an increase of model complexity and number of model parameters, which, in practice, makes such models difficult to apply to experimental data. There are several previous attempts to simplify existing models. For example, Wagenmakers et al. (2007) proposed a simplified version of the DDM by assuming that there is no between-trial variability, and a further simplified DDM proposed by Grasman et al. (2009) additionally assumes the starting point of the integrator is not biased toward any alternative. These simplified models can directly estimate the DDM parameters from analytical solutions without a parameter-fitting procedure.

More recently, Brown and Heathcote (2008) proposed a linear ballistic accumulator (LBA) model of choice decisions (see Brown and Heathcote, 2005a for a non-linear version of the model). The LBA model has been applied to many choice tasks including perceptual discrimination (Forstmann et al., 2008, 2010a,b; Ho et al., 2009), absolute identification (Brown and Heathcote, 2008), lexical decisions (Donkin and Heathcote, 2009), and saccadic eye movements (Ludwig et al., 2009; Farrell et al., 2010). Similar to the LCA model, the LBA model assumes each integrator integrates evidence supporting one alternative and hence can be applied to NAFC tasks, but with two major simplifications. First, the integrators are independent (no mutual inhibition) and have no leakage (no decay). Second, the integration process within each trial is linear and deterministic (i.e., ballistic), omitting the within-trial variability in momentary evidence. These two assumptions greatly simplify the model dynamics and hence the LBA model has analytical solutions for RT distributions and response accuracies for NAFC tasks. This is a significant advantage in terms of computational complexity as one can estimate the model parameters without using Monte Carlo simulations. However, the strong assumptions inevitably introduce limitations. Because the integration process is assumed to be linear and deterministic, the LBA model cannot distinguish evidence arriving at different times over a trial, and hence it is not straightforward to apply the LBA model when accounting for primacy and recency effects, or any task paradigms that deliberately introduce temporal uncertainty within a trial (Usher and McClelland, 2001; Huk and Shadlen, 2005; Tsetsos et al., 2011).

Decision-making models can be used to isolate decision components (e.g., boundary and drift rates), from which estimated model parameters can infer experimental data collected from different sources, such as fMRI or EEG/MEG signals. This model-based approach provides an invaluable way of linking latent decision processes predicted by the accumulator models with their implementations in large neural populations, and not surprisingly has attracted increasing interest over the last few years (Philiastides et al., 2006; Philiastides and Sajda, 2007; Forstmann et al., 2008, 2010b; Ho et al., 2009; Ratcliff et al., 2009; Kayser et al., 2010a,b; Wenzlaff et al., 2011). It is worth noting that all models can be used for this purpose, although simpler models are often employed due to less computational complexity.

**Figure 3:**
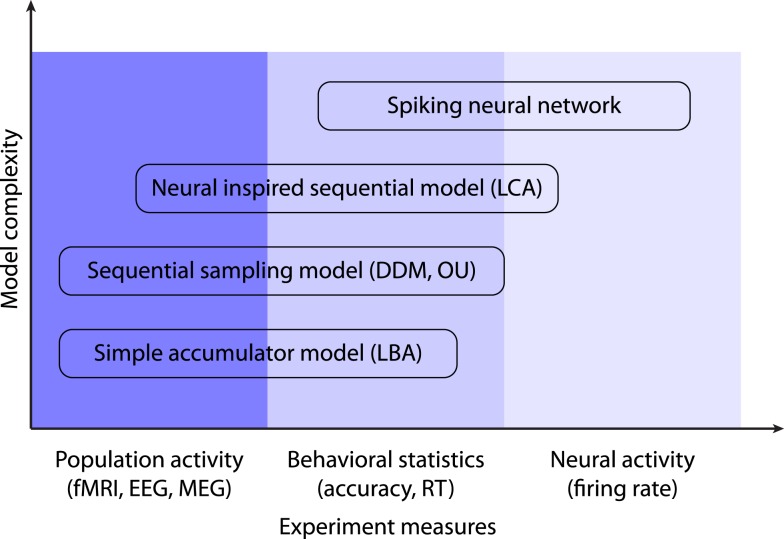
**The complexity and generality of the decision-making models**. All models are capable of capturing basic behavioral statistics such as the RT and the response accuracy. The simple accumulator models and the sequential sampling models are suitable to describe the congregate activity of large neural populations (e.g., fMRI or EEG/MEG signals). The most complex model (i.e., the spiking neural network) can be used to account for dynamics of neural circuits.

However, models at a highly abstract level (e.g., the DDM and the LBA model) are not sufficient to address some more fundamental questions of decision-making, such as the neural mechanism of slow ramping activity in LIP neurons during RDM tasks, or the mechanisms of decay and inhibition in neural integrators. The answers to these questions require more detailed models at the level of single neurons (the LCA model provides a middle ground in neural plausibility between single neuron models and the DDM). Wang (2002) proposed a biophysically based spiking neuron model for perceptual decision-making. For the RDM task with two alternatives, the model assumes two LIP neural populations supporting each alternative. Instead of mutual inhibition in the LCA model, all neurons from different populations project to a common pool of inhibitory neurons, which then inhibits each population via feedback inhibitory connections. Wang (2002) proposed that evidence integration over a long timescale (on the order of several hundred milliseconds to over 1 s), as assumed by most sequential sampling models, could be realistically carried out by neural populations with recurrent excitatory connections mediated by NMDA receptors at a very short timescale (on the order of less than 100 ms). This model has been demonstrated to successfully account for the activity of LIP neurons as well as behavioral performance in the RDM tasks (Wong and Wang, 2006; Wong et al., 2007), and has recently been applied to multiple alternative decision tasks (Furman and Wang, 2008). However, although the biophysical model is important for understanding the neural mechanisms of decision processes, due to the model complexity and the large number of model parameters it could be difficult to use such a specialized model as an exploratory tool for other decision tasks, or to search through the parameter space to fit the model to RT distributions. Smith and McKenzie (2011) recently proposed a simplified version of Wang’s (2002) model that overcomes these difficulties. In their minimal recurrent loop model, evidence is represented by Poisson shot noise processes (Smith, 2010) and evidence integration for each alternative is represented by the superposition of Poisson processes, resembling the essential statistical features of the reverberation loops in Wang’s model. The model provides a theoretical account of how diffusive-like evidence integration at an abstract level naturally emerges from the spike densities in the recurrent loops. Further, at a cost of two more free parameters, the minimal recurrent loop model can fit the RT distributions and associated choice accuracies almost equally well as the DDM (Smith and McKenzie, 2011), suggesting that the model offers a promising balance between biological plausibility and generality to predict experimental data. In summary, decision models at different levels of complexity could be useful to capture experimental data obtained from different modalities (Figure 3), and empirical researchers should choose an appropriate model that suits their research questions.

## Theoretical Considerations of Evidence Boundaries

### Boundary mechanisms

**Figure 4:**
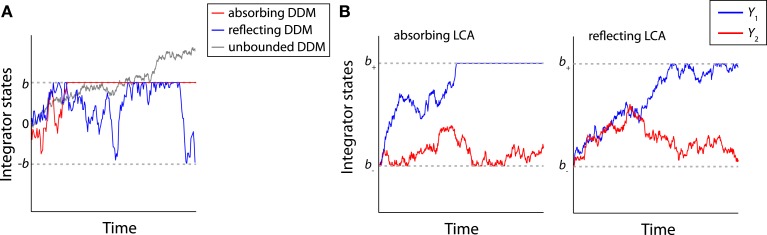
**Time course of the integrators of the DDM and LCA model with boundaries**. **(A)** Examples of trajectories of the absorbing (red), reflecting (blue) and unbounded (gray) DDM. Two boundaries (±*b*) are indicated by the gray dashed lines. **(B)** Examples of trajectories of the absorbing (left panel) and reflecting (right panel) LCA models. The lower boundary (*b*_−_) and the upper boundary (*b*_+_) boundaries are indicated by the gray dashed lines.

All the sequential sampling models discussed above describe a diffusion-like evidence integration during the decision process (Brown and Holmes, 2001; Brown et al., 2005). However they need to be bundled with evidence boundaries that constrain accumulation. This section examines evidence boundaries according to two different but not mutually exclusive definitions: (1) evidence boundaries that determine the amount of accumulated evidence required to make a decision (i.e., the decision boundaries), and (2) evidence boundaries that act as barriers to the amount of accumulated evidence (Figure 4A).

The first type of evidence boundary, hereafter referred to as the *absorbing* boundary, provides an evidence criterion or threshold for the termination state of an integration process, and assumes a decision is made once accumulated evidence supporting one alternative reaches the boundary. The absorbing boundary is necessary for modeling tasks that require subjects to implement a self-initiated stopping rule (e.g., in the IC paradigm) and hence it has been widely used by many models in the choice RT modeling literature (Ratcliff, 1988, 2006; Gomez et al., 2007).

The second type of evidence boundary introduces biologically inspired constraints that limit the amount of accumulated evidence. Early decision models did not explicitly constrain activity of integrators (Ratcliff, 1978), which raised theoretical and practical concerns to the validity of the models. The theoretical concern is that unconstrained integrators imply a possibility of an unlimited amount of evidence being maintained by the model (Figure 4A). For example, in the TC paradigm, the integrator state of the DDM has infinite mean and variance as *T_c_* approaches infinity (see Eq. 1). For the LCA model, unconstrained integrators further imply the possibility that model activation may become negative due to mutual inhibition. Unlimited or negative activations are undesirable for a biologically plausible model, because neural integrators cannot exceed certain values due to intrinsic limitations of biological neurons. Their activity should also be non-negative. These constraints need to be satisfied before attempting to extend abstract models to qualitatively account for neural firing rate patterns during the decision process (Usher and McClelland, 2001; Ratcliff et al., 2003a; Huk and Shadlen, 2005; Ditterich, 2006; Purcell et al., 2010).

The practical concern is that models with unconstrained integrators may not fit experimental data well. In the TC paradigm, the ER of the DDM with an unconstrained integrator diminishes to zero for a large decision time *T_c_* (without between-trial variability), and hence the model predicts that subjects can achieve arbitrarily small ER even for difficult tasks. Nevertheless, it is known that humans cannot achieve 100% accuracy even for large *T_c_* (Meyer et al., 1988; Usher and McClelland, 2001). Furthermore, negative activation in the LCA model may result in abnormal model predictions. Bogacz et al. (2007) showed that in a multi-alternative decision task, if the inputs to an LCA model favor only a small subset of possible alternatives, integrators favoring irrelevant choices (i.e., those that do not receive inputs) would become negative and send uninformative positive evidence via mutual inhibition to the relevant competing integrators (i.e., those receiving inputs). As a result the LCA model without truncation of negative activation may select inferior alternatives in value-based decisions (Usher and McClelland, 2004; Usher et al., 2008), and provide qualitatively poorer fits to experimental data than the models with non-negative evidence only (Leite and Ratcliff, 2010). The same problem also exists in models with feed-forward inhibitory connections (van Ravenzwaaij et al., 2012).

One way to introduce constraints is to transform the integrator state through a non-linear activation function (Brown and Holmes, 2001; Usher and McClelland, 2001; Brown et al., 2005), or to assume high-level baseline activity for avoiding non-negative activations (van Ravenzwaaij et al., 2012). A simpler approach, without losing the explicit nature and tractability of a linear system and yet offering a good approximation of the non-linear activation functions, is to introduce explicit evidence boundaries to existing models. This type of boundary is hereafter referred to as the *reflecting boundary* (Diederich, 1995; Bogacz et al., 2007; Zhang et al., 2009; Zhang and Bogacz, 2010a; Smith and McKenzie, 2011). The reflecting boundary only constrains the maximum or minimum amount of evidence that can be presented by an integrator (much as a non-linear activation function provides cutoffs at high or low activations), but unlike the absorbing boundary, reaching a reflecting boundary does not terminate the integration process (Figure 4A).

Both types of boundary mechanisms have been applied to various decision models (Ratcliff, 2006; Bogacz et al., 2007; Zhang et al., 2009; Zhang and Bogacz, 2010a; Tsetsos et al., 2011; van Ravenzwaaij et al., 2012). The decision models with boundaries are hereafter referred to as *bounded*, and the models without a boundary as *unbounded*. For the DDM and the OU model, when there is no bias toward either alternative, two symmetric absorbing or reflecting boundaries (±*b*) can be imposed to limit the integrator’s activity (Figure 4A). For simplicity, the terms absorbing DDM and absorbing OU model are used when the two absorbing boundaries apply to the models, and the reflecting DDM and reflecting OU model when referring to models with two reflecting boundaries. For an LCA model with multiple integrators, if one assumes that integrators cannot have arbitrarily large or negative values, then two boundary conditions need to be applied to each integrator (Figure 4B). First, each integrator requires one lower boundary *b_−_* at zero to constrain the minimum activity to be non-negative (Bogacz et al., 2007). This lower boundary needs to be a reflecting boundary, since otherwise the model may not render a decision (i.e., if the lower boundary is absorbing, activities of all integrators could be fixed at the boundary). Second, each integrator requires one upper boundary *b*_+_ (*b*_+_ > 0) to limit the maximum activity. The upper boundary *b*_+_ could be either absorbing or reflecting. The LCA model with an absorbing boundary at *b*_+_ is referred to as the absorbing LCA model, and the model with a reflecting boundary at *b*_+_ as the reflecting LCA model. Table 1 summarizes the bounded decision models discussed above and their properties.

**Table 1:** **Properties of the sequential sampling models with and without boundaries**.

		Primacy	Recency	Optimality	TC paradigm	IC paradigm
DDM	Unbound	–	–	Optimal	✓	✓
	Absorbing	✓	–	–	✓	✓
	Reflecting	–	✓	–	✓	–
OU	Unbound	λ > 0	λ < 0	λ = 0	✓	✓
	Absorbing	Various	λ < 0	λ < 0	✓	✓
	Reflecting	λ > 0	Various	λ > 0	✓	–
LCA	Unbound	*w* > *k*	*w* < *k*	*k* = *w*	✓	✓
	Lower-bound	*w* > *k*	*w* < *k*	Unknown	✓	✓
	Absorbing	Unknown	Unknown	*w* < *k*	✓	✓
	Reflecting	Unknown	Unknown	*w* > *k*	✓	–

*The lower-bound LCA model refers to the LCA model that has only lower reflecting boundary at zero but no upper boundary*.

It is worth noting that models with absorbing boundaries provide a unified account for both IC and TC paradigms (Ratcliff and McKoon, 2008), because contact with absorbing boundaries induces a decision. In contrast, models with pure reflecting boundaries require an external criterion to stop (e.g., decision deadline *T_c_*), and hence they are only for the TC paradigm but cannot account for the IC paradigm. Although the pure reflecting model may be criticized for its lack of generality, it is necessary to consider the models with pure reflecting boundaries together alongside models with absorbing boundaries in order to illustrate some complementary properties of the two types of boundary. First, absorbing boundaries, together with the reflecting boundaries, provide a simple solution for primacy and recency effects in different models (see Primacy and Recency Effects). Second, the two types of boundary could characterize different decision strategies in the TC paradigm (Zhang and Bogacz, 2010a). The absorbing boundary implies that subjects make their choice before the response deadline (i.e., once the absorbing boundary is reached) and withhold their decision. The reflecting boundary implies that subjects continuously hesitate between the choices even when sufficient evidence is available (i.e., when the reflecting boundary is reached) and may change their decision later. Whether subjects adopt one of the two strategies, or are able to switch between the two (see Tsetsos et al., 2012), would be an interesting question for future research.

### Primacy and recency effects

**Figure 5:**
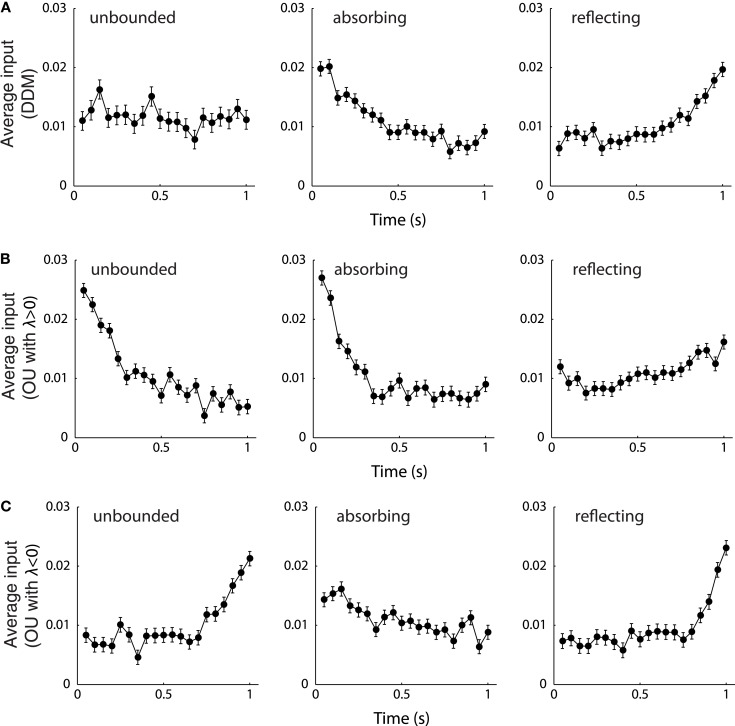
**The primacy and recency effects of the DDM and OU model**. **(A)** The bounded and unbounded DDM. **(B)** The bounded and unbounded OU models with λ > 0. **(C)** The bounded and unbounded OU model with λ < 0. All the models were simulated with μ = 0.71 s^−1^, σ = 1 s^−1^, *b* = 0.47, and *T_c_* = 1 s. The growth-decay parameter of the OU models was set to λ = 5.5 **(B)** and λ = −5.5 **(C)**. In each panel, the model was simulated for 10,000 trials, and the sensory evidence from all correct trials was recorded and averaged. The data points show the means and standard errors of the sensory evidence at every time step. For μ > 0, a larger averaged input indicates that the sensory evidence at that time point has, on average, a larger influence on the final choice, and a smaller averaged input indicates that the choice depends to a lesser extent on the evidence at that time. Figure modified from Zhang and Bogacz (2010a).

The unbounded DDM integrates evidence independent of the current integrator state (Eq. 1), and hence the model implies that influence of sensory evidence on the final choice does not depend on the timing of its occurrence (i.e., neither primacy nor recency). One recent study suggests that the DDM can account for primacy and recency effects by introducing the two types of boundaries (Zhang et al., 2009). For the absorbing DDM, if a boundary is reached before decision time, the preferred decision is determined and only evidence occurring prior to the boundary hit contributes to the integration process, indicating a primacy effect. For the reflecting DDM, each boundary hit results in a partial loss of evidence, since the integrator does not fully integrate momentary evidence that would otherwise exceed the boundary. As a result, the momentary evidence arriving earlier is partially lost and on average a decision depends to a greater extent on later evidence, indicating a recency effect (Figure 5A). A further study indicates that the primacy/recency effects introduced by the two types of boundaries can coincide and interact with the effects introduced by the growth-decay parameter λ in a bounded OU model (Zhang and Bogacz, 2010a). If the boundary and λ provide the same effect, the joint primacy/recency effect of the bounded OU model is maintained. On the contrary, the joint effect of the bounded OU model is weakened or canceled if λ and the boundary present opposite effects (Figures 5B,C). For example, for λ > 0 (primacy effect), an OU model with absorbing boundaries (also the primacy effect) will also exhibit a strong primacy effect, but an OU model with reflecting boundaries will show a weaker effect. There is as yet no study systematically reporting primacy and recency effects in the bounded LCA model. Given the close relationship between LCA model and OU model, one may expect that the primacy/recency effects of bounded LCA model are jointly determined by the type of boundary and the value of inhibition and decay parameters. Recent studies (Tsetsos et al., 2011, 2012) demonstrates that the LCA model with only lower reflecting boundary demonstrates a strong primacy effect when the inhibition is large relative to the decay (*w* > *k*), and a recency effect when the inhibition is small relative to the decay (*w* < *k*), consistent with results obtained from the unbounded LCA model.

This section has shown that primacy and recency effects can be readily produced by evidence boundaries or their interactions with other model parameters. Nevertheless, existing experimental data is insufficient to demonstrate the strength of these effects in the way predicted by the models. An ideal paradigm to systematically investigate and differentiate these effects would be a decision task using time-varying evidence, which favors one alternative early in a trial and another alternative later in a trial. However, the interpretation of results from such an experiment would need to proceed cautiously in case of potential confounds. First, if non-stationary stimuli extends for a long period of time (as in the expanded judgment paradigm, see Pietsch and Vickers, 1997), the observed primacy/recency effects may be to some extent associated with additional attention or working memory processes. Second, if non-stationarity in the evidence is apparent to subjects, they may consciously change their decision strategy. Several studies on rapid perceptual decisions avoided these methodological problems by using carefully designed paradigms. Brown and Heathcote (2005b) presented strong prime stimuli for a very short time and used a metacontrast mask to ensure subjects did not consciously aware the non-stationarity. They showed that early evidence is weighted less in a perceptual decision task (i.e., the integration is leaky), but the leakage quickly decreased with practice. In Usher and McClelland’s (2001) study, primacy/recency effects were tested with fast visual streams of alternating letters lasting for only 256 ms. They randomly mixed shorter trials with non-stationary evidence and longer trials with constant evidence. Such a design encouraged subjects to estimate the entire sequence of the non-stationary evidence, because making decisions on only a fraction of early evidence would result in low performance on longer trials. Their results suggest a general recency effect with strong individual differences, although the source of the large between-subject variability has not yet been identified.

### Performance of the bounded decision-making models

Several studies have reported significant improvements in model fit by introducing evidence boundaries. Ratcliff (2006) fitted data for the DDM and the LCA model from a categorization task in which subjects were required to decide whether the number of dots on the screen is large or small. The absorbing DDM and absorbing LCA model provide much better fits than the unbounded models, in particular for the TC paradigm with very short or long decision times. Another study showed that for a shape discrimination task (Usher and McClelland, 2001), the behavioral data is more likely to have been fitted by the bounded DDM than by the unbounded OU model (Zhang et al., 2009). Leite and Ratcliff (2010) showed that the LCA model with zero reflecting boundary produced better fits to the RT distributions than the unbounded model in perceptual decision tasks with different number of alternatives. Zhang et al. (2009) observed that for a given set of model parameters, the ER of the absorbing and reflecting DDM are identical at any decision time. Therefore, although the two types of boundary influence the model dynamics, and weight the order of the momentary evidence in different ways, the two bounded DDMs can fit the experimental data from the TC paradigm equally well. A similar equality between absorbing and reflecting OU models has also been observed (Zhang and Bogacz, 2010a).

**Figure 6:**
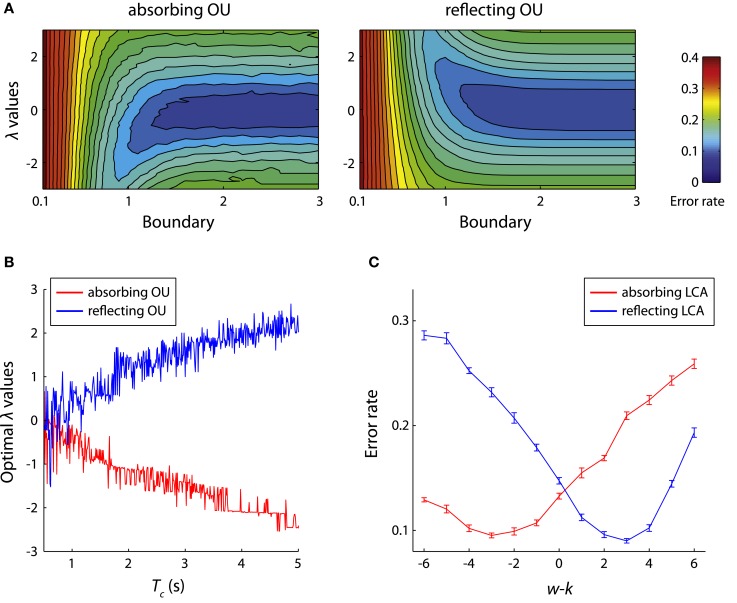
**Performance of the bounded models**. **(A)** The error rates of the absorbing (left) and reflecting (right) OU models in the TC paradigm. The bounded OU models are simulated with the following parameters: λ in (−3, 3) with step 0.1, *b* in (0.1, 3) with step 0.1, μ = σ = 1 s^−1^, and *T_c_* = 1 s. The contour plots illustrate the mean error rates of the bounded OU models estimated from 10,000 simulations for each possible parameter combinations. Figure modified from Zhang and Bogacz (2010a). **(B)** The estimated optimal λ values of the absorbing and reflecting OU models that yield minimum error rate for different *T_c_* varying from 0.5 to 5 s. Figure modified from Zhang and Bogacz (2010a). **(C)** The error rates of the bounded LCA model. The models were simulated with parameters: μ_1_ = 5.41 s^−1^, μ_2_ = 4 s^−1^, σ = 1 s^−1^, *b*_+_ = 1.5, *b*_−_ = 0, and *T_c_* = 3 s. The sum of decay and inhibition was fixed at *w* + *k* = 6, while their difference changed from −6 to 6.

The successful applications of the bounded models promote us to consider how different types of evidence boundaries may affect the models’ performance. For the IC paradigm, adding lower reflecting boundaries at zero generally decreases mean RT of the LCA model for a given ER, and this change is more significant for decision tasks with multiple alternatives (Bogacz et al., 2007; Leite and Ratcliff, 2010). Increasing the upper boundary in the absorbing LCA model, or the distance between the two boundaries in the absorbing DDM and absorbing OU model, leads to an increase in the mean and variance of RT distributions (Wagenmakers et al., 2005) and a decrease of ER (i.e., trading speed for accuracy, see Fast Boundary Modulation: Speed–Accuracy Tradeoff). For the TC paradigm, the bounded DDM has an asymptotic accuracy as *T_c_* increases, which is consistent with experimental observations (Meyer et al., 1988; Usher and McClelland, 2001). Increasing boundary separation in the bounded DDM monotonically decrease the ER for a given decision time, until the boundary is sufficiently large that the integrator can barely reach the boundary before *T_c_*, and under this condition the bounded DDM model is equivalent to the unbounded DDM (Zhang et al., 2009; Leite and Ratcliff, 2010). Interestingly, the relationship between the evidence boundary and the ER is not monotonic in the bounded OU model (Zhang and Bogacz, 2010a). For the OU model with a negative λ value, a finite absorbing boundary yields lower ER than the unbounded OU model. In contrast, a finite reflecting boundary lowers the ER for the OU model with a positive λ value (Figure 6A). Simulation results suggested that as *T_c_* increases, the value of λ that yields the lowest ER decreases for the absorbing OU model and increases for the reflecting OU model (Figure 6B). This relationship can be explained by the joint primacy/recency effects from the boundary and the λ value of the bounded OU model (see Primacy and Recency Effects). Recall that the optimal decision strategy, as suggested by the SPRT and NPT, would be to equally weight the momentary evidence received at different time points (i.e., no primacy or recency effects). The bounded OU model approximates to the optimal strategy when the primacy/recency effects introduced by the boundary and λ are balanced. That is, the absorbing OU model needs to be coupled with negative λ and the reflecting OU model needs to be coupled with positive λ. The relative strengths of the primacy/recency effects introduced by the boundary and λ values deserve further research.

The findings from one-dimensional bounded models provide clues to the understanding of performance of the bounded LCA model. Recall that the unbounded LCA model implements the optimal decision strategy when the decay and inhibition are balanced (*w* = *k*), i.e., when the LCA model is reduced to the DDM. Bogacz et al. (2007) showed that the balance of decay and inhibition does not optimize the performance of the bounded LCA model in the TC paradigm. Instead, by decreasing inhibition relative to decay (*w* < *k*) the absorbing LCA model can achieve lower ER. Conversely, the reflecting LCA model has lower ER when inhibition is larger than decay (*w* > *k*; Figure 6C). The symmetric relationship between the absorbing and reflecting LCA models is analogous to that of bounded OU models with positive and negative λ. Therefore it is possible that the bounded LCA model can be reduced to the bounded OU model for certain parameters (cf. van Ravenzwaaij et al., 2012). Bogacz et al. (2007) also suggest that by limiting the integrator stages to be non-negative, the absorbing LCA model can approximates the asymptotically optimal decision strategy (Draglia et al., 1999; Dragalin et al., 2000) for multiple alternative tasks (Bogacz and Gurney, 2007).

## Neural Implementation of Decision Boundary

How is the decision boundary realized in neural circuits? In the minimal recurrent loop model by Smith and McKenzie (2011), the decision boundary is implemented by an interaction between the recurrent loops and separate decision neurons. The decision neurons receive spiking inputs from the recurrent loops that represent the accumulated evidence. A decision is rendered as soon as the membrane potential of one decision neuron reaches a threshold. This mechanism predicts a causal link between the firing of decision neurons and overt actions. But an important question remains: where in the brain is the decision boundary implemented?

One possibility is that the decision boundary is implemented within neural integrators, namely the *local* *hypothesis*. Wong and Wang (2006) studied a simplified version of the biologically based model of Wang (2002) by using mean-field theory. Their analysis showed that if neural integrators are mediated by recurrent excitatory connections between spiking neurons, the dynamics of neural integrators may contain multiple stable attractor states, which act as implicit decision boundaries to terminate integration processes. This model successfully accounts for psychophysical data and LIP neural activity in RDM tasks (Wong and Wang, 2006; Wong et al., 2007). However, previous studies using the RDM task or other visual discrimination tasks have identified putative neural integrators in the FEF (Hanes and Schall, 1996; Schall and Thompson, 1999; Schall, 2002), the SC (Basso and Wurtz, 1998; Ratcliff et al., 2003a), and the DLPFC (Kim and Shadlen, 1999; Domenech and Dreher, 2010), which exhibit activity patterns similar to LIP neurons. A recent study showed that the inferior frontal sulcus is also likely to integrate evidence from multiple sensory modalities (Noppeney et al., 2010). Therefore, multiple neural integrators may coexist in different brain regions and may be simultaneously functioning during a decision process, though we do not know whether the neural integrators across different regions are independent or are more likely to interact with each other. If the local hypothesis is correct, it is yet not clear whether observed boundary crossing in one integrator region has a causal role in rendering a decision, or could merely reflect terminal integration in other integrator regions. Further experiments testing the activity of neural integrators in predefined regions under different decision tasks are necessary to confirm this hypothesis.

An alternative possibility, the *central hypothesis*, proposes that detection of boundary crossing is implemented by a central neural circuit outside integrator regions, rather than an intrinsic property of neural integrators. This hypothesis predicts that a central circuit is capable of detecting boundary crossing in integrators within different regions. One potential component of the central circuit is the basal ganglia (BG) because of its unique anatomy. First, the two BG input nuclei, the striatum and the subthalamic nucleus, receive direct inputs from multiple cortical regions including the LIP, FEF, and DLPFC (Smith et al., 1998; Hikosaka et al., 2000; Nakano et al., 2000). Second, most BG nuclei are organized in separate somatotopic areas representing different body parts, and each broad somatotopic area is further subdivided into functionally defined parallel channels, based upon specific movements of an individual body part (Alexander et al., 1986, 1990; Parent and Hazrati, 1995). Therefore the BG can access a number of information sources from the cortex and control complex motor responses, which make the BG important loci of action selection, reinforcement learning, and motor control (Karabelas and Moschovakis, 1985; Graybiel et al., 1994; Gurney et al., 2001a,b; Frank et al., 2004; Samejima et al., 2005). Lo and Wang (2006) proposed that detection of boundary crossing is implemented through a BG-SC pathway. By default the BG output nuclei send tonic inhibition (Hopkins and Niessen, 1976; Francois et al., 1984; Karabelas and Moschovakis, 1985) to downstream motor areas (e.g., the SC) to suppress any saccadic response. When the activity of a neural integrator (e.g., LIP neurons) is large enough, the striatum inhibits BG output nuclei and hence releases inhibition to the SC. The boundary crossing is then detected by burst neurons (Munoz and Wurtz, 1995) in the SC by an all-or-nothing burst signal. Bogacz and Gurney (2007) showed that the BG is necessary for the brain to implement asymptotically optimal decision strategy for NAFC tasks. Nevertheless, although Lo and Wang (2006) demonstrated that the central hypothesis can be implemented by the BG-SC circuit, the model relies on the unique burst property of the SC neurons to detect boundary crossing, which is primarily associated with eye movements. It is not clear whether the same mechanism can be applied to decision tasks requiring other response modalities (e.g., Ho et al., 2009), or tasks which require subjects to withhold their responses before a response signal (i.e., the TC paradigm).

Taken together, although convincing data exists for the presence of neural integrators in the cortex, current findings are inconclusive regarding the neural implementation of decision boundaries. Part of the difficulty in investigating the boundary mechanism is that decision neurons may exhibit task-modulated ramping activity that is similar to neural integrators, if there exists positive feedback connections between the decision neurons and the integrators (Simen, 2012). As a result the two processes may be indistinguishable solely by the observation of ramping activity from neural recording data.

## Effects of Boundary Changes

The decision boundary is usually assumed to be under subjective control. On one hand, the decision boundary should be stable in regards to sensory evidence, enabling subjects to respond consistently when faced with similar environments or goals. The stability of the decision boundary is evident from the fact that in both IC and TC versions of the RDM tasks, LIP neurons attain the same level of activity before saccadic responses, independent of motion coherence (Shadlen and Newsome, 2001; Roitman and Shadlen, 2002). On the other hand, the decision boundary may also exhibit a certain degree of flexibility, allowing subjects to tailor their responses on demand, or accounting for changes in some internally driven factors. This section reviews psychological and physiological factors that could be modulated by changes in the decision boundary at different time scales.

### Fast boundary modulation: Speed–accuracy tradeoff

**Figure 7:**
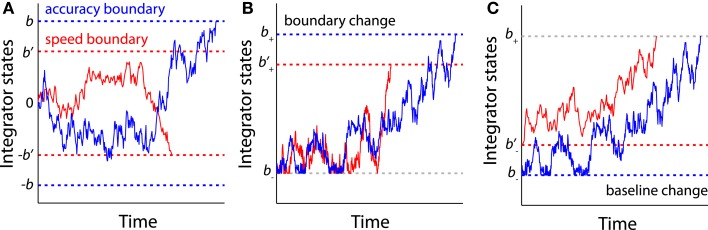
**The sequential sampling models account for SAT**. **(A)** For the models with a single integrator (e.g., the DDM and the OU model), increasing the distance between two boundaries (blue boundaries ±*b*) leads to slow but accurate decisions, while decreasing the boundary distance (red boundaries ±*b*’) leads to fast but risky decisions. **(B)** For the models with multiple integrator (e.g., the LCA model), the SAT can be accounted for by changes in the upper boundary (*b*_+_ and *b*’_+_). **(C)** The SAT can also be accounted for by changes in the lower baseline activity (*b*_−_ and $b_{-}^{\prime }$).

The change in decision boundary provides a straightforward account of the speed–accuracy tradeoff (SAT) effect that is often observed in decision-making tasks (Schouten and Bekker, 1967; Wickelgren, 1977; Luce, 1986; Franks et al., 2003; Chittka et al., 2009). For the DDM and the OU model (Figure 7A), decreasing the distance between two decision boundaries reduces the amount of accumulated evidence prior to a decision, leading to fast but error-prone responses. Conversely, increasing the distance between boundaries leads to slow but accurate decisions. For the LCA model or other models that have multiple integrators (e.g., the LBA model), the SAT can be manipulated by changing either the upper boundary (Figure 7B) or the lower baseline activity at the beginning of the trial (Figure 7C) (Bogacz et al., 2010b). Behavioral studies suggest that subjects can effectively trade speed for accuracy when instructed to respond as accurately as possible, or vice versa when instructed to respond as quickly as possible, and the behavioral differences between speed and accuracy instructions can be explained by a change of decision boundaries in the DDM (Palmer et al., 2005; Ratcliff, 2006; Ratcliff and McKoon, 2008). In a similar attempt to study SAT using the LBA model, Forstmann et al. (2008) observed that SAT in the RDM task can be best accounted for by a change in the decision boundary, not by changes of the drift rate or other model parameters. It has been suggested that humans can set the SAT to maximize the reward rate (producing the most correct decisions in a given period of time) by learning the optimal decision boundaries through feedback (Simen et al., 2006, 2009; Bogacz et al., 2010a; Starns and Ratcliff, 2010; Balci et al., 2011). Furthermore, impairments in the optimization of the SAT in neuropsychiatric patients with impulsive behaviors, such as attention-deficit hyperactivity disorder, has been associated with maladaptive regulation of the decision boundary in perceptual tasks (Mulder et al., 2010).

**Figure 8:**
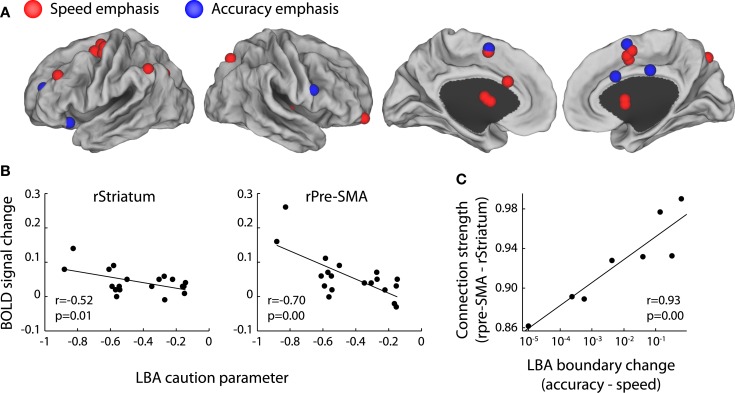
**The neural correlates of SAT**. **(A)** Brain regions associated with the SAT are projected onto a cortical surface using Caret software (Van Essen et al., 2001). The foci represent the coordinates of the peak voxels reported by four fMRI studies (Forstmann et al., 2008; Ivanoff et al., 2008; van Veen et al., 2008; van Maanen et al., 2011). All the studies manipulated the SAT of perceptual decision tasks by speed emphasis or accuracy emphasis. The red foci illustrate increased BOLD response with speed emphasis and the blue foci illustrate increased BOLD response with accuracy emphasis. **(B)** In the RDM task, the BOLD response increases in the right Pre-SMA and the right Striatum in the speed versus the accuracy condition. These BOLD response changes are associated with decreases in the response caution parameter, which is quantified by boundary changes in the LBA model. Figure modified from Forstmann et al. (2008). **(C)** The strength of structural connections between the Pre-SMA and the Striatum in individual subjects correlate with the changes of the LBA decision boundary between the speed and the accuracy condition. Figure modified from Forstmann et al. (2010a).

Can we consider the SAT as a signature for identifying neural correlates of decision boundaries? Several recent fMRI studies reveal brain regions associated with the SAT, including the SMA, the pre-SMA, the anterior cingulate cortex, the striatum, and the DLPFC (Forstmann et al., 2008; Ivanoff et al., 2008; van Veen et al., 2008; Blumen et al., 2011; van Maanen et al., 2011; for review, see Bogacz et al., 2010b; Figure 8A). Using a model-based fMRI analysis, Forstmann et al. (2008) showed that the extent of response facilitation for the speed condition in the RDM task, as quantified by a decrease of the decision boundary in the LBA model, correlated with BOLD response increase in the pre-SMA and striatum between the speed and the accuracy conditions (Figure 8B). Further studies suggest that the strength of structural connectivity between the two regions predicts the amount of boundary change in individual subjects (Forstmann et al., 2010a, 2011; Figure 8C). These results support the central hypothesis that the BG circuit is involved in controlling the decision boundary (Lo and Wang, 2006; Bogacz et al., 2010b).

Nevertheless, some concerns remain regarding the causal role of decision boundary in SAT. First, an emphasis on speed may be associated with other cognitive processes (Rinkenauer et al., 2004). For example, some studies have proposed that the integration process is coupled with an urgency signal that increases as a function of time (Churchland et al., 2008; Cisek et al., 2009). The urgency signal effectively lowers the decision boundary as time elapses (Ditterich, 2006), and the SAT can be attributed to a change in strength of the urgency signal. Second, some models predict that SAT is in fact controlled by the distance between the boundary and baseline (Figure 7C). Hence emphasizing speed or accuracy may modulate the decision boundary, baseline, or a combination of the two (Bogacz et al., 2010b; Simen, 2012). In particular, decreasing decision boundary is equivalent to increasing baseline activations in the LBA model. Recent fMRI studies suggest that the SAT is more likely to modulate baseline activity in the medial frontal cortex (pre-SMA and SMA), as these regions exhibit a greater BOLD response in the speed instruction compared to the accuracy instruction. Other studies suggest that SAT may modulate a decision boundary in the lateral PFC, where the speed instruction is associated with decreased BOLD responses (Ivanoff et al., 2008; Wenzlaff et al., 2011). However, it is possible that the aforementioned cortical areas do not directly change the decision boundary or baseline, but provide a control signal that modulates striatal activity (Bogacz et al., 2010b). In a recent neurophysiological study (Heitz and Schall, 2011), monkeys were trained to trade accuracy for speed in a visual search task. Fitting the behavioral data with the LBA model showed that the speed instruction can be accounted for by a decrease in the decision boundary. Interestingly, speed instruction led to an increased baseline activity as well as an increased presaccadic activity in the FEF, suggesting that the neural implementation of SAT likely involves multiple processes, rather than a single boundary or baseline change predicted by psychological models.

### Slow boundary modulation: Perceptual learning and aging

It is well-known that practice can improve performance in many perceptual tasks, resulting in higher accuracy and shorter RTs (Logan, 1992; Heathcote et al., 2000). Traditional approaches usually quantify learning effects as changes in the mean accuracy or RT. Several recent studies have attempted to decompose component processes mediating perceptual learning by using sequential sampling models. Petrov et al. (2011) fitted the DDM to behavioral data from a fine motion-discrimination task and showed that learning effects across multiple training sessions are mainly associated with an increase in drift rate and a decrease in non-decision time (see also Dutilh et al., 2009). This result is consistent with previous findings that learning facilitates neural representation of task-relevant features by tuning neural selectivity in the sensory areas (Gilbert et al., 2001; Yang and Maunsell, 2004; Kourtzi and DiCarlo, 2006; Raiguel et al., 2006; Kourtzi, 2010; Zhang et al., 2010). Other studies suggest that extensive training also leads to a significant reduction in the boundary distance in the DDM (Ratcliff et al., 2006; Dutilh et al., 2009; Liu and Watanabe, 2011). Using the RDM task, Liu and Watanabe (2011) investigated the learning effect across different days and showed that training without feedback decreases the decision boundary in the DDM and also increases drift rate. Dutilh et al. (2009) proposed that a dual process (changes in both boundary and drift rate) is necessary to account for the noticeable decrease in RT even after the improvement in accuracy saturates during training. The involvement of boundary reduction in perceptual learning is supported by experimental findings that perceptual learning may not only change sensory representation, but also enhance the decision process in intraparietal regions (Law and Gold, 2008; Zhang and Kourtzi, 2010). Further research combining a modeling approach with multiple imaging sessions over the course of training may reveal how learning and feedback modulate sensory representation and decision processes during perceptual decisions.

While training may improve the ability of subjects to make faster decisions in perceptual decision tasks and result in a lower decision boundary, one primary finding in aging is that RTs in cognitive tasks increase as people age, and this generalized slowing is sometimes coupled with impairments in accuracy (Cerella, 1985, 1991; Fisk and Warr, 1996; Salthouse, 1996). Recent studies have employed the DDM with behavioral data to identify the effects of aging in a number of choice tasks (Ratcliff et al., 2001, 2003b, 2004b, 2007; Thapar et al., 2003; Spaniol et al., 2006). A consistent observation is that slowing in older adults can be explained by two factors: an increase in the decision boundary and a prolongation of non-decision time. The decision boundary increase in aging suggests that older subjects are more cautious in making decisions compared with younger subjects (Ratcliff et al., 2006; Starns and Ratcliff, 2010). This age-dependent change in the decision boundary may be due to structural limits in pre-SMA and striatal connectivity (Forstmann et al., 2011) or functional impairments in the striatum (Kühn et al., 2011) in the aging brain. These findings are consistent with the central hypothesis that the striatum is involved in modulating decision boundaries.

## Discussion

This article has reviewed recent developments that shed light on the effects and mechanisms of evidence boundaries. Theoretically, boundaries shape the dynamics of decision processes in two aspects. First, the evidence boundary provides an ecological function to constrain the evidence needed for rendering a decision, since the nervous system cannot process an unlimited amount of information. Second, the evidence boundary provides a mechanistic function to determine the termination of a decision process. The necessity of the evidence boundary is not limited to a specific model, but is a common feature shared by different sequential sampling models and other accumulator models (e.g., the LBA model), independent of the model structures. Empirically, the presence of evidence boundary is evident from behavioral, neurophysiological and neuroimaging data. Existing findings suggest that evidence boundaries remains stable to changes in the external environment (e.g., sensory information), but may vary systematically with some internal factors (e.g., speed or accuracy emphasis, practice, or aging). Whether acting on its own, or interacting with other decision-related processes, boundaries play a crucial role in the formation of decisions. Therefore boundary mechanisms provide a window into understanding the cognitive processes associated with choice behavior.

Despite the increasing number of recent studies examining the evidence boundary, we are still far from a complete picture of its functions and neural implementations. Here I suggest several directions that merit further investigation. First, among decision models that implement the integration-to-boundary mechanism, it is not clear to what extent the effect of a boundary depend on the specific structure of the models. For example, if for a given dataset the DDM predicts a change in the boundary between two experimental conditions, or a correlation between the estimated boundary and cognitive assessment scores (e.g., Ratcliff et al., 2008), would we reach the same conclusion if using the LCA model or the LBA model? van Ravenzwaaij and Oberauer (2009) suggested that boundaries estimated from different sequential sampling models are generally consistent, but do not necessarily correspond with those estimated from the LBA model (cf. Donkin et al., 2011). Such discrepancies between models need be considered if researchers plan to estimate boundary changes from experimental data, or use estimated model parameters to guide subsequent neuroimaging analysis.

Psychological models conceptualize the evidence boundary as a unitary representation. The neural implementation of evidence boundaries is likely to be more sophisticated and remains to be determined (see Simen et al., 2011; Smith and McKenzie, 2011 for recent attempts to bridge the gap between the two). The existing findings favor the central hypothesis over the local hypothesis, but we do not yet fully understand the causal relationship between the activity of the BG nuclei and the changes of the boundary. Studies discussed in this article suggests that boundary changes can occur at different time scales, ranging from a few seconds during which the SAT can be effectively adapted, to a few days during which it is necessary to modulate the boundary through extensive training and feedback. Hence if a central neural circuit exists for the detection of boundary crossing, this system is likely to be affected by different underlying control signals, but we do not know how and where in the brain the control signals for boundary changes are encoded. A related question is how the evidence boundary may be affected by aging or neurodegenerative diseases. Could these long-term factors alter control signals that modulate the boundary, or directly act upon the neural circuits that implement the boundary? Answering these questions will require researchers to combine established modeling approaches with comprehensive neuroimaging protocols.

Finally, existing findings suggest that the integration-to-boundary process governs a broad range of cognitive tasks (Gold and Shadlen, 2007). An important direction for future research is to investigate the effects of boundaries in choice tasks other than perceptual decisions. One example is interval timing estimation, in which subjects produce or estimate a specific duration (Church and Deluty, 1977; Roberts, 1981; Rakitin et al., 1998; Macar et al., 1999; Allan and Gerhardt, 2001). A variant of the DDM has recently been proposed for interval timing (Simen et al., 2011). The model assumes a single integrator with variable drift rate representing elapsed time at different durations and a constant decision boundary. A fixed boundary predicted by the model is supported by experimental findings that slow cortical potentials measured in the pre-SMA/SMA, which have been interpreted as a signature of time accumulation process, show no amplitude difference between different interval times (Elbert et al., 1991; Pfeuty et al., 2005; Kononowicz and van Rijn, 2011; Ng et al., 2011). Another example is voluntary action decision, which require subjects to make selections between actions that have no differential sensory attributes or action outcomes (Brass and Haggard, 2008; Haggard, 2008; Soon et al., 2008; Andersen and Cui, 2009; Roskies, 2010). Recent studies propose that during the formation of voluntary decisions the intention of selecting each action gradually builds up in independent integrators until the winning integrators reaches the boundary and renders the decision (Zhang et al., 2012). This hypothesis is supported by observations of a progressive rise in the readiness potential and neural activity in the medial prefrontal cortex before consciously aware of voluntary actions (Libet, 1985; Sirigu et al., 2004; Fried et al., 2011). These findings from different types of cognitive tasks suggest that the brain may encode the evidence boundary as a common currency for perceptual information, subjective intention, or individual preference (e.g., Chib et al., 2009; Krajbich et al., 2010) to guide behavioral responses, depending on the context of the task. An intriguing possibility is that evidence boundaries associated with different cognitive tasks may be mediated by the same neural implementation. This generic implementation provides a potential bridge between behavioral and neural data to regulate the formation and initiation of complex behavior.

## Conflict of Interest Statement

The author declares that the research was conducted in the absence of any commercial or financial relationships that could be construed as a potential conflict of interest.

## Footnotes

^1^The term “decision boundary” is referred to the type of evidence boundary that directly affects the termination of the decision. The tem “evidence boundary” is referred to all types of boundaries that limit the accumulation process. See Section “Theoretical Considerations of Evidence Boundaries” for a detailed discussion.

## References

[B1] Alexander G. E. Crutcher M. D. DeLong M. R. (1990). Basal ganglia-thalamocortical circuits: parallel substrates for motor, oculomotor, “prefrontal” and “limbic” functions. Prog. Brain Res. 85, 119–146 10.1016/S0079-6123(08)62678-3 2094891

[B2] Alexander G. E. DeLong M. R. Strick P. L. (1986). Parallel organization of functionally segregated circuits linking basal ganglia and cortex. Annu. Rev. Neurosci. 9, 357–381 10.1146/annurev.ne.09.030186.002041 3085570

[B3] Allan L. G. Gerhardt K. (2001). Temporal bisection with trial referents. Percept. Psychophys. 63, 524–540 10.3758/BF03194418 11414139

[B4] Andersen R. A. Cui H. (2009). Intention, action planning, and decision making in parietal-frontal circuits. Neuron 63, 568–583 10.1016/j.neuron.2009.08.028 19755101

[B5] Balci F. Simen P. Niyogi R. Saxe A. Hughes J. A. Holmes P. Cohen J. D. (2011). Acquisition of decision making criteria: reward rate ultimately beats accuracy. Atten. Percept. Psychophys. 73, 640–657 10.3758/s13414-010-0049-7 21264716 PMC3383845

[B6] Barnard G. A. (2007). Sequential tests in industrial statistics. J. R. Stat. Soc. 8, 1–26

[B7] Basso M. A. Wurtz R. H. (1998). Modulation of neuronal activity in superior colliculus by changes in target probability. J. Neurosci. 18, 7519–7534 9736670 10.1523/JNEUROSCI.18-18-07519.1998 PMC6793246

[B8] Blumen H. M. Gazes Y. Habeck C. Kumar A. Steffener J. Rakitin B. C. Stern Y. (2011). Neural networks associated with the speed-accuracy tradeoff: evidence from the response signal method. Behav. Brain Res. 224, 397–402 10.1016/j.bbr.2011.06.004 21699922 PMC3159733

[B9] Bogacz R. (2007). Optimal decision-making theories: linking neurobiology with behaviour. Trends Cogn. Sci. (Regul. Ed.) 11, 118–125 10.1016/j.tics.2006.12.006 17276130

[B10] Bogacz R. Brown E. Moehlis J. Holmes P. Cohen J. D. (2006). The physics of optimal decision making: a formal analysis of models of performance in two-alternative forced-choice tasks. Psychol. Rev. 113, 700–765 10.1037/0033-295X.113.4.700 17014301

[B11] Bogacz R. Gurney K. (2007). The basal ganglia and cortex implement optimal decision making between alternative actions. Neural Comput. 19, 442–477 10.1162/neco.2007.19.2.442 17206871

[B12] Bogacz R. Hu P. T. Holmes P. J. Cohen J. D. (2010a). Do humans produce the speed-accuracy trade-off that maximizes reward rate? Q. J. Exp. Psychol. (Hove) 63, 863–891 10.1080/17470210903091643 19746300 PMC2908414

[B13] Bogacz R. Wagenmakers E.-J. Forstmann B. U. Nieuwenhuis S. (2010b). The neural basis of the speed-accuracy tradeoff. Trends Neurosci. 33, 10–16 10.1016/j.tins.2009.09.002 19819033

[B14] Bogacz R. Usher M. Zhang J. McClelland J. L. (2007). Extending a biologically inspired model of choice: multi-alternatives, nonlinearity and value-based multidimensional choice. Philos. Trans. R. Soc. Lond. B Biol. Sci. 362, 1655–1670 10.1098/rstb.2007.2059 17428774 PMC2440778

[B15] Born R. T. Bradley D. C. (2005). Structure and function of visual area MT. Annu. Rev. Neurosci. 28, 157–189 10.1146/annurev.neuro.26.041002.131052 16022593

[B16] Brass M. Haggard P. (2008). The what, when, whether model of intentional action. Neuroscientist 14, 319–325 10.1177/1073858408317417 18660462

[B17] Britten K. Shadlen M. Newsome W. Movshon J. (1992). The analysis of visual motion: a comparison of neuronal and psychophysical performance. J. Neurosci. 12, 4745–4765 1464765 10.1523/JNEUROSCI.12-12-04745.1992 PMC6575768

[B18] Britten K. H. Newsome W. T. Shadlen M. N. Celebrini S. Movshon J. A. (1996). A relationship between behavioral choice and the visual responses of neurons in macaque MT. Vis. Neurosci. 13, 87–100 10.1017/S095252380000715X 8730992

[B19] Britten K. H. Shadlen M. N. Newsome W. T. Movshon J. A. (1993). Responses of neurons in macaque MT to stochastic motion signals. Vis. Neurosci. 10, 1157–1169 10.1017/S0952523800010269 8257671

[B20] Brown E. Gao J. Holmes P. Bogacz R. (2005). Simple neural networks that optimize decisions. Int. J. Bifurcat. Chaos 15, 803–826 10.1142/S0218127405012478

[B21] Brown E. Holmes P. (2001). Modelling a simple choice task: stochastic dynamics of mutually inhibitory neural groups. Stochast. Dynam. 1, 159–191 10.1142/S0219493701000102

[B22] Brown S. Heathcote A. (2005a). A ballistic model of choice response time. Psychol. Rev. 112, 117–128 10.1037/0033-295X.112.1.117 15631590

[B23] Brown S. Heathcote A. (2005b). Practice increases the efficiency of evidence accumulation in perceptual choice. J. Exp. Psychol. Hum. Percept. Perform. 31, 289–298 10.1037/0096-1523.31.2.289 15826231

[B24] Brown S. D. Heathcote A. (2008). The simplest complete model of choice response time: linear ballistic accumulation. Cogn. Psychol. 57, 153–178 10.1016/j.cogpsych.2007.12.002 18243170

[B25] Busemeyer J. (2002). Survey of decision field theory. Math. Soc. Sci. 43, 345–370 10.1016/S0165-4896(02)00016-1

[B26] Busemeyer J. R. Jessup R. K. Johnson J. G. Townsend J. T. (2006). Building bridges between neural models and complex decision making behaviour. Neural Netw. 19, 1047–1058 10.1016/j.neunet.2006.05.043 16979319

[B27] Busemeyer J. R. Townsend J. T. (1993). Decision field theory: a dynamic-cognitive approach to decision making in an uncertain environment. Psychol. Rev. 100, 432–459 10.1037/0033-295X.100.3.432 8356185

[B28] Cerella J. (1985). Information processing rates in the elderly. Psychol. Bull. 98, 67–83 10.1037/0033-2909.98.1.67 4034819

[B29] Cerella J. (1991). Age effects may be global, not local: comment on Fisk and Rogers (1991). J. Exp. Psychol. Gen. 120, 215–223 10.1037/0096-3445.120.2.215 1830612

[B30] Chib V. S. Rangel A. Shimojo S. O’Doherty J. P. (2009). Evidence for a common representation of decision values for dissimilar goods in human ventromedial prefrontal cortex. J. Neurosci. 29, 12315–12320 10.1523/JNEUROSCI.2575-09.2009 19793990 PMC6666137

[B31] Chittka L. Skorupski P. Raine N. E. (2009). Speed-accuracy tradeoffs in animal decision making. Trends Ecol. Evol. (Amst.) 24, 400–407 10.1016/j.tree.2009.02.010 19409649

[B32] Church R. M. Deluty M. Z. (1977). Bisection of temporal intervals. J. Exp. Psychol. Anim. Behav. Process. 3, 216–228 10.1037/0097-7403.3.3.216 881613

[B33] Churchland A. K. Kiani R. Shadlen M. N. (2008). Decision-making with multiple alternatives. Nat. Neurosci. 11, 693–702 10.1038/nn.2123 18488024 PMC2453226

[B34] Cisek P. Puskas G. A. El-Murr S. (2009). Decisions in changing conditions: the urgency-gating model. J. Neurosci. 29, 11560–11571 10.1523/JNEUROSCI.1844-09.2009 19759303 PMC6665752

[B35] Diederich A. (1995). Intersensory facilitation of reaction time: evaluation of counter and diffusion coactivation models. J. Math. Psychol. 39, 197–215 10.1006/jmps.1995.1020

[B36] Diederich A. (1997). Dynamic stochastic models for decision making under time constraints. J. Math. Psychol. 41, 260–274 10.1006/jmps.1997.1167 9325121

[B37] Ditterich J. (2006). Stochastic models of decisions about motion direction: behavior and physiology. Neural Netw. 19, 981–1012 10.1016/j.neunet.2006.05.042 16952441

[B38] Ditterich J. (2010). A comparison between mechanisms of multi-alternative perceptual decision making: ability to explain human behavior, predictions for neurophysiology, and relationship with decision theory. Front. Neurosci. 4:184 10.3389/fnins.2010.00184 21152262 PMC2999395

[B39] Ditterich J. Mazurek M. E. Shadlen M. N. (2003). Microstimulation of visual cortex affects the speed of perceptual decisions. Nat. Neurosci. 6, 891–898 10.1038/nn1094 12858179

[B40] Domenech P. Dreher J.-C. (2010). Decision threshold modulation in the human brain. J. Neurosci. 30, 14305–14317 10.1523/JNEUROSCI.2371-10.2010 20980586 PMC6634811

[B41] Donkin C. Brown S. Heathcote A. Wagenmakers E.-J. (2011). Diffusion versus linear ballistic accumulation: different models but the same conclusions about psychological processes? Psychon. Bull. Rev. 18, 61–69 10.3758/s13423-010-0022-4 21327360 PMC3042112

[B42] Donkin C. Heathcote A. (2009). “Non-decision time effects in the lexical decision task,” in Proceedings of the 31st Annual Conference of the Cognitive Science Society, eds Taatgen N. A. van Rijn H. (Austin: Cognitive Science Society), 2902–2907

[B43] Dosher B. A. (1976). The retrieval of sentences from memory: a speed-accuracy study. Cogn. Psychol. 8, 291–310 10.1016/0010-0285(76)90009-8

[B44] Dosher B. A. (1984). Discriminating preexperimental (semantic) from learned (episodic) associations: a speed-accuracy study. Cogn. Psychol. 16, 519–555 10.1016/0010-0285(84)90019-7

[B45] Dragalin V. P. Tartakovsky A. G. Veeravalli V. V. (2000). Multihypothesis sequential probability ratio tests. II. Accurate asymptotic expansions for the expected sample size. IEEE Trans. Inf. Theory 46, 1366–1383 10.1109/18.850677

[B46] Draglia V. P. Tartakovsky A. G. Veeravalli V. V. (1999). Multihypothesis sequential probability ratio tests. I. Asymptotic optimality. IEEE Trans. Inf. Theory 45, 2448–2461 10.1109/18.796383

[B47] Dutilh G. Vandekerckhove J. Tuerlinckx F. Wagenmakers E.-J. (2009). A diffusion model decomposition of the practice effect. Psychon. Bull. Rev. 16, 1026–1036 10.3758/16.6.1026 19966251

[B48] Edwards W. (1965). Optimal strategies for seeking information: models for statistics, choice reaction times, and human information processing. J. Math. Psychol. 2, 312–329 10.1016/0022-2496(65)90007-6

[B49] Elbert T. Ulrich R. Rockstroh B. Lutzenberger W. (1991). The processing of temporal intervals reflected by CNV-like brain potentials. Psychophysiology 28, 648–655 10.1111/j.1469-8986.1991.tb01009.x 1816592

[B50] Estes W. K. (1955). Statistical theory of spontaneous recovery and regression. Psychol. Rev. 62, 145–154 10.1037/h0046888 14371893

[B51] Farrell S. Ludwig C. J. H. Ellis L. A. Gilchrist I. D. (2010). Influence of environmental statistics on inhibition of saccadic return. Proc. Natl. Acad. Sci. U.S.A. 107, 929–934 10.1073/pnas.0913026107 20080778 PMC2818969

[B52] Fisk J. E. Warr P. (1996). Age and working memory: the role of perceptual speed, the central executive, and the phonological loop. Psychol. Aging 11, 316–323 10.1037/0882-7974.11.2.316 8795060

[B53] Forstmann B. U. Anwander A. Schäfer A. Neumann J. Brown S. Wagenmakers E.-J. Bogacz R. Turner R. (2010a). Cortico-striatal connections predict control over speed and accuracy in perceptual decision making. Proc. Natl. Acad. Sci. U.S.A. 107, 15916–15920 10.1073/pnas.1004932107 20733082 PMC2936628

[B54] Forstmann B. U. Brown S. Dutilh G. Neumann J. Wagenmakers E.-J. (2010b). The neural substrate of prior information in perceptual decision making: a model-based analysis. Front. Hum. Neurosci. 4:40 10.3389/fnhum.2010.00040 20577592 PMC2889713

[B55] Forstmann B. U. Dutilh G. Brown S. Neumann J. von Cramon D. Y. Ridderinkhof K. R. Wagenmakers E.-J. (2008). Striatum and pre-SMA facilitate decision-making under time pressure. Proc. Natl. Acad. Sci. U.S.A. 105, 17538–17542 10.1073/pnas.0805903105 18981414 PMC2582260

[B56] Forstmann B. U. Tittgemeyer M. Wagenmakers E.-J. Derrfuss J. Imperati D. Brown S. (2011). The speed-accuracy tradeoff in the elderly brain: a structural model-based approach. J. Neurosci. 31, 17242–17249 10.1523/JNEUROSCI.0309-11.2011 22114290 PMC6623864

[B57] Francois C. Percheron G. Yelnik J. (1984). Localization of nigrostriatal, nigrothalamic and nigrotectal neurons in ventricular coordinates in macaques. Neuroscience 13, 61–76 10.1016/0306-4522(84)90259-8 6387531

[B58] Frank M. J. Seeberger L. C. O’Reilly R. C. (2004). By carrot or by stick: cognitive reinforcement learning in parkinsonism. Science 306, 1940–1943 10.1126/science.1102941 15528409

[B59] Franks N. R. Dornhaus A. Fitzsimmons J. P. Stevens M. (2003). Speed versus accuracy in collective decision making. Proc. Biol. Sci. 270, 2457–2463 10.1098/rsbl.2003.0047 14667335 PMC1691524

[B60] Fried I. Mukamel R. Kreiman G. (2011). Internally generated preactivation of single neurons in human medial frontal cortex predicts volition. Neuron 69, 548–562 10.1016/j.neuron.2010.11.045 21315264 PMC3052770

[B61] Furman M. Wang X.-J. (2008). Similarity effect and optimal control of multiple-choice decision making. Neuron 60, 1153–1168 10.1016/j.neuron.2008.12.003 19109918 PMC2633638

[B62] Gilbert C. D. Sigman M. Crist R. E. (2001). The neural basis of perceptual learning. Neuron 31, 681–697 10.1016/S0896-6273(01)00424-X 11567610

[B63] Gold J. I. Shadlen M. N. (2001). Neural computations that underlie decisions about sensory stimuli. Trends Cogn. Sci. (Regul. Ed.) 5, 10–16 10.1016/S1364-6613(00)01567-9 11164731

[B64] Gold J. I. Shadlen M. N. (2007). The neural basis of decision making. Annu. Rev. Neurosci. 30, 535–574 10.1146/annurev.neuro.29.051605.113038 17600525

[B65] Gomez P. Ratcliff R. Perea M. (2007). A model of the go/no-go task. J. Exp. Psychol. Gen. 136, 389–413 10.1037/0096-3445.136.3.389 17696690 PMC2701630

[B66] Grasman R. P. P. P. Wagenmakers E.-J. van der Maas H. L. J. (2009). On the mean and variance of response times under the diffusion model with an application to parameter estimation. J. Math. Psychol. 53, 55–68 10.1016/j.jmp.2009.01.006

[B67] Graybiel A. Aosaki T. Flaherty A. Kimura M. (1994). The basal ganglia and adaptive motor control. Science 265, 1826–1831 10.1126/science.8091209 8091209

[B68] Gurney K. Prescott T. J. Redgrave P. (2001a). A computational model of action selection in the basal ganglia. I. A new functional anatomy. Biol. Cybern. 84, 401–410 10.1007/PL00007985 11417052

[B69] Gurney K. Prescott T. J. Redgrave P. (2001b). A computational model of action selection in the basal ganglia. II. Analysis and simulation of behaviour. Biol. Cybern. 84, 411–423 10.1007/PL00007985 11417053

[B70] Haggard P. (2008). Human volition: towards a neuroscience of will. Nat. Rev. Neurosci. 9, 934–946 10.1038/nrn2497 19020512

[B71] Hanes D. P. Schall J. D. (1996). Neural control of voluntary movement initiation. Science 274, 427–430 10.1126/science.274.5286.427 8832893

[B72] Hanks T. D. Ditterich J. Shadlen M. N. (2006). Microstimulation of macaque area LIP affects decision-making in a motion discrimination task. Nat. Neurosci. 9, 682–689 10.1038/nn1683 16604069 PMC2770004

[B73] Heath R. (1992). A general nonstationary diffusion model for two-choice decision-making. Math. Soc. Sci. 23, 283–309 10.1016/0165-4896(92)90044-6

[B74] Heathcote A. Brown S. Mewhort D. J. K. (2000). The power law repealed: the case for an exponential law of practice. Psychon. Bull. Rev. 7, 185–207 10.3758/BF03212979 10909131

[B75] Heekeren H. R. Marrett S. Ungerleider L. G. (2008). The neural systems that mediate human perceptual decision making. Nat. Rev. Neurosci. 9, 467–479 10.1038/nrn2374 18464792

[B76] Heitz R. P. Schall J. D. (2011). “Neural basis of speed-accuracy trade-off in frontal eye field,” in Abstracts of the Society for Neuroscience Annual Meeting 2011 (Washington, DC: Society for Neuroscience).

[B77] Hikosaka O. Takikawa Y. Kawagoe R. (2000). Role of the basal ganglia in the control of purposive saccadic eye movements. Physiol. Rev. 80, 953–978 10893428 10.1152/physrev.2000.80.3.953

[B78] Ho T. C. Brown S. Serences J. T. (2009). Domain general mechanisms of perceptual decision making in human cortex. J. Neurosci. 29, 8675–8687 10.1523/JNEUROSCI.5175-08.2009 19587274 PMC2719543

[B79] Hopkins D. A. Niessen L. W. (1976). Substantia nigra projections to the reticular formation, superior colliculus and central gray in the rat, cat and monkey. Neurosci. Lett. 2, 253–259 10.1016/0304-3940(76)90156-7 19604767

[B80] Huk A. C. Shadlen M. N. (2005). Neural activity in macaque parietal cortex reflects temporal integration of visual motion signals during perceptual decision making. J. Neurosci. 25, 10420–10436 10.1523/JNEUROSCI.4684-04.2005 16280581 PMC6725829

[B81] Ivanoff J. Branning P. Marois R. (2008). fMRI evidence for a dual process account of the speed-accuracy tradeoff in decision-making. PLoS ONE 3, e2635 10.1371/journal.pone.0002635 18612380 PMC2440815

[B82] Karabelas A. B. Moschovakis A. K. (1985). Nigral inhibitory termination on efferent neurons of the superior colliculus: an intracellular horseradish peroxidase study in the cat. J. Comp. Neurol. 239, 309–329 10.1002/cne.902390305 2995462

[B83] Kayser A. S. Buchsbaum B. R. Erickson D. T. D’Esposito M. (2010a). The functional anatomy of a perceptual decision in the human brain. J. Neurophysiol. 103, 1179–1194 10.1152/jn.00364.2009 20032247 PMC2887630

[B84] Kayser A. S. Erickson D. T. Buchsbaum B. R. D’Esposito M. (2010b). Neural representations of relevant and irrelevant features in perceptual decision making. J. Neurosci. 30, 15778–15789 10.1523/JNEUROSCI.3163-10.2010 21106817 PMC3020592

[B85] Kiani R. Hanks T. D. Shadlen M. N. (2008). Bounded integration in parietal cortex underlies decisions even when viewing duration is dictated by the environment. J. Neurosci. 28, 3017–3029 10.1523/JNEUROSCI.4761-07.2008 18354005 PMC6670720

[B86] Kim J. N. Shadlen M. N. (1999). Neural correlates of a decision in the dorsolateral prefrontal cortex of the macaque. Nat. Neurosci. 2, 176–185 10.1038/5739 10195203

[B87] Kononowicz T. W. van Rijn H. (2011). Slow potentials in time estimation: the role of temporal accumulation and habituation. Front. Integr. Neurosci. 5:10 10.3389/fnint.2011.00048 21949505 PMC3171873

[B88] Kourtzi Z. (2010). Visual learning for perceptual and categorical decisions in the human brain. Vision Res. 50, 433–440 10.1016/j.visres.2009.09.025 19818361

[B89] Kourtzi Z. DiCarlo J. J. (2006). Learning and neural plasticity in visual object recognition. Curr. Opin. Neurobiol. 16, 152–158 10.1016/j.conb.2006.03.012 16563736

[B90] Krajbich I. Armel C. Rangel A. (2010). Visual fixations and the computation and comparison of value in simple choice. Nat. Neurosci. 13, 1292–1298 10.1038/nn.2635 20835253

[B91] Kühn S. Schmiedek F. Schott B. Ratcliff R. Heinze H.-J. Düzel E. Lindenberger U. Lövden M. (2011). Brain areas consistently linked to individual differences in perceptual decision-making in younger as well as older adults before and after training. J. Cogn. Neurosci. 23, 2147–2158 10.1162/jocn.2010.21564 20807055

[B92] Laming D. R. J. (1968). Information Theory of Choice-Reaction Times. Oxford: Academic Press

[B93] Law C.-T. Gold J. I. (2008). Neural correlates of perceptual learning in a sensory-motor, but not a sensory, cortical area. Nat. Neurosci. 11, 505–513 10.1038/nn2070 18327253 PMC2424192

[B94] Lehmann E. (1959). Testing Statistical Hypotheses. New York: Wiley

[B95] Leite F. P. Ratcliff R. (2010). Modeling reaction time and accuracy of multiple-alternative decisions. Atten. Percept. Psychophys. 72, 246–273 10.3758/APP.72.1.246 20045893 PMC2805113

[B96] Libet B. (1985). Unconscious cerebral initiative and the role of conscious will in voluntary action. Behav. Brain Sci. 8, 529–539 10.1017/S0140525X00045155

[B97] Link S. W. (1975). The relative judgment theory of two choice response time. J. Math. Psychol. 12, 114–135 10.1016/0022-2496(75)90053-X

[B98] Link S. W. Heath R. A. (1975). A sequential theory of psychological discrimination. Psychometrika 40, 77–105 10.1007/BF02291481

[B99] Liu C. C. Watanabe T. (2011). Accounting for speed-accuracy tradeoff in perceptual learning. Vision Res. 61, 107–114 10.1016/j.visres.2011.09.007 21958757 PMC3288618

[B100] Lo C.-C. Wang X.-J. (2006). Cortico-basal ganglia circuit mechanism for a decision threshold in reaction time tasks. Nat. Neurosci. 9, 956–963 10.1038/nn1722 16767089

[B101] Logan G. D. (1992). Shapes of reaction-time distributions and shapes of learning curves: a test of the instance theory of automaticity. J. Exp. Psychol. Learn Mem. Cogn. 18, 883–914 10.1037/0278-7393.18.5.883 1402715

[B102] Luce R. D. (1986). Response Times: Their Role in Inferring Elementary Mental Organization. New York: Oxford University Press

[B103] Ludwig C. J. H. Farrell S. Ellis L. A. Gilchrist I. D. (2009). The mechanism underlying inhibition of saccadic return. Cogn. Psychol. 59, 180–202 10.1016/j.cogpsych.2009.04.002 19520369 PMC2734060

[B104] Macar F. Vidal F. Casini L. (1999). The supplementary motor area in motor and sensory timing: evidence from slow brain potential changes. Exp. Brain Res. 125, 271–280 10.1007/s002210050683 10229018

[B105] Maunsell J. H. Van Essen D. C. (1983). Functional properties of neurons in middle temporal visual area of the macaque monkey. I. Selectivity for stimulus direction, speed, and orientation. J. Neurophysiol. 49, 1127–1147 6864242 10.1152/jn.1983.49.5.1127

[B106] Mazurek M. E. Roitman J. D. Ditterich J. Shadlen M. N. (2003). A role for neural integrators in perceptual decision making. Cereb. Cortex 13, 1257–1269 10.1093/cercor/bhg097 14576217

[B107] Mcmillen T. Holmes P. (2006). The dynamics of choice among multiple alternatives. J. Math. Psychol. 50, 30–57 10.1016/j.jmp.2005.10.003

[B108] Meyer D. E. Irwin D. E. Osman A. M. Kounios J. (1988). The dynamics of cognition and action: mental processes inferred from speed-accuracy decomposition. Psychol. Rev. 95, 183–237 10.1037/0033-295X.95.3.340 3375399

[B109] Mulder M. J. Bos D. Weusten J. M. H. van Belle J. van Dijk S. C. Simen P. van Engeland H. Durston S. (2010). Basic impairments in regulating the speed-accuracy tradeoff predict symptoms of attention-deficit/hyperactivity disorder. Biol. Psychiatry 68, 1114–1119 10.1016/j.biopsych.2010.07.031 20926067

[B110] Munoz D. P. Wurtz R. H. (1995). Saccade-related activity in monkey superior colliculus. I. Characteristics of burst and buildup cells. J. Neurophysiol. 73, 2313–2333 7666141 10.1152/jn.1995.73.6.2313

[B111] Nakano K. Kayahara T. Tsutsumi T. Ushiro H. (2000). Neural circuits and functional organization of the striatum. J. Neurol. 247, V1–V15 10.1007/PL00007778 11081799

[B112] Newsome W. Pare E. (1988). A selective impairment of motion perception following lesions of the middle temporal visual area (MT). J. Neurosci. 8, 2201–2211 3385495 10.1523/JNEUROSCI.08-06-02201.1988 PMC6569328

[B113] Newsome W. T. Britten K. H. Movshon J. A. (1989). Neuronal correlates of a perceptual decision. Nature 341, 52–54 10.1038/341052a0 2770878

[B114] Neyman J. Pearson E. S. (1933). On the problem of the most efficient tests of statistical hypotheses. Philos. Trans. R. Soc. Lond. A 231, 289–337 10.1098/rsta.1933.0009

[B115] Ng K. K. Tobin S. Penney T. B. (2011). Temporal accumulation and decision processes in the duration bisection task revealed by contingent negative variation. Front. Integr. Neurosci. 5:77 10.3389/fnint.2011.00077 22144952 PMC3225905

[B116] Niwa M. Ditterich J. (2008). Perceptual decisions between multiple directions of visual motion. J. Neurosci. 28, 4435–4445 10.1523/JNEUROSCI.5564-07.2008 18434522 PMC6670944

[B117] Noppeney U. Ostwald D. Werner S. (2010). Perceptual decisions formed by accumulation of audiovisual evidence in prefrontal cortex. J. Neurosci. 30, 7434–7446 10.1523/JNEUROSCI.0455-10.2010 20505110 PMC6632395

[B118] Palmer J. Huk A. C. Shadlen M. N. (2005). The effect of stimulus strength on the speed and accuracy of a perceptual decision. J. Vis. 5, 376–404 10.1167/5.5.1 16097871

[B119] Papoulis A. (1977). Signal Analysis. New York: McGraw-Hill

[B120] Parent A. Hazrati L.-N. (1995). Functional anatomy of the basal ganglia. I. The cortico-basal ganglia-thalamo-cortical loop. Brain Res. Rev. 20, 91–127 10.1016/0165-0173(94)00007-C 7711769

[B121] Petrov A. A. Van Horn N. M. Ratcliff R. (2011). Dissociable perceptual-learning mechanisms revealed by diffusion-model analysis. Psychon. Bull. Rev. 18, 490–497 10.3758/s13423-011-0079-8 21394547

[B122] Pfeuty M. Ragot R. Pouthas V. (2005). Relationship between CNV and timing of an upcoming event. Neurosci. Lett. 382, 106–111 10.1016/j.neulet.2005.02.067 15911131

[B123] Philiastides M. G. Ratcliff R. Sajda P. (2006). Neural representation of task difficulty and decision making during perceptual categorization: a timing diagram. J. Neurosci. 26, 8965–8975 10.1523/JNEUROSCI.1655-06.2006 16943552 PMC6675324

[B124] Philiastides M. G. Sajda P. (2007). EEG-informed fMRI reveals spatiotemporal characteristics of perceptual decision making. J. Neurosci. 27, 13082–13091 10.1523/JNEUROSCI.3540-07.2007 18045902 PMC6673396

[B125] Pietsch A. Vickers D. (1997). Memory capacity and intelligence: novel techniques for evaluating rival models of a fundamental information-processing mechanism. J. Gen. Psychol. 124, 229–339 10.1080/00221309709595520 9438970

[B126] Pike A. R. (1966). Stochastic models of choice behaviour: response probabilities and latencies of finite Markov chain systems. Br. J. Math. Stat. Psychol. 19, 15–32 10.1111/j.2044-8317.1966.tb00351.x 5939142

[B127] Ploran E. J. Nelson S. M. Velanova K. Donaldson D. I. Petersen S. E. Wheeler M. E. (2007). Evidence accumulation and the moment of recognition: dissociating perceptual recognition processes using fMRI. J. Neurosci. 27, 11912–11924 10.1523/JNEUROSCI.3522-07.2007 17978031 PMC6673371

[B128] Purcell B. A. Heitz R. P. Cohen J. Y. Schall J. D. Logan G. D. Palmeri T. J. (2010). Neurally constrained modeling of perceptual decision making. Psychol. Rev. 117, 1113–1143 10.1037/a0020311 20822291 PMC2979343

[B129] Raiguel S. Vogels R. Mysore S. G. Orban G. A. (2006). Learning to see the difference specifically alters the most informative V4 neurons. J. Neurosci. 26, 6589–6602 10.1523/JNEUROSCI.0457-06.2006 16775147 PMC6674023

[B130] Rakitin B. C. Gibbon J. Penney T. B. Malapani C. Hinton S. C. Meck W. H. (1998). Scalar expectancy theory and peak-interval timing in humans. J. Exp. Psychol. Anim. Behav. Process. 24, 15–33 10.1037/0097-7403.24.1.15 9438963

[B131] Ratcliff R. (1978). A theory of memory retrieval. Psychol. Rev. 85, 59–108 10.1037/0033-295X.85.2.59

[B132] Ratcliff R. (1988). Continuous versus discrete information processing modeling accumulation of partial information. Psychol. Rev. 95, 238–255 10.1037/0033-295X.95.3.385 3375400

[B133] Ratcliff R. (2002). A diffusion model account of response time and accuracy in a brightness discrimination task: fitting real data and failing to fit fake but plausible data. Psychon. Bull. Rev. 9, 278–291 10.3758/BF03196302 12120790

[B134] Ratcliff R. (2006). Modeling response signal and response time data. Cogn. Psychol. 53, 195–237 10.1016/j.cogpsych.2005.10.002 16890214 PMC2397556

[B135] Ratcliff R. Cherian A. Segraves M. (2003a). A comparison of macaque behavior and superior colliculus neuronal activity to predictions from models of two-choice decisions. J. Neurophysiol. 90, 1392–1407 10.1152/jn.01049.2002 12761282

[B136] Ratcliff R. Thapar A. McKoon G. (2003b). A diffusion model analysis of the effects of aging on brightness discrimination. Percept. Psychophys. 65, 523–535 10.3758/BF03194580 12812276 PMC1360154

[B137] Ratcliff R. Gomez P. McKoon G. (2004a). A diffusion model account of the lexical decision task. Psychol. Rev. 111, 159–182 10.1037/0033-295X.111.1.159 14756592 PMC1403837

[B138] Ratcliff R. Thapar A. McKoon G. (2004b). A diffusion model analysis of the effects of aging on recognition memory. J. Mem. Lang. 50, 408–424 10.1016/j.jml.2003.11.00216981012

[B139] Ratcliff R. McKoon G. (2008). The diffusion decision model: theory and data for two-choice decision tasks. Neural Comput. 20, 873–922 10.1162/neco.2008.12-06-420 18085991 PMC2474742

[B140] Ratcliff R. Philiastides M. G. Sajda P. (2009). Quality of evidence for perceptual decision making is indexed by trial-to-trial variability of the EEG. Proc. Natl. Acad. Sci. U.S.A. 106, 6539–6544 10.1073/pnas.0812589106 19342495 PMC2672543

[B141] Ratcliff R. Rouder J. N. (1998). Modeling response times for two-choice decisions. Psychol. Sci. 9, 347–356 10.1111/1467-9280.00067

[B142] Ratcliff R. Rouder J. N. (2000). A diffusion model account of masking in two-choice letter identification. J. Exp. Psychol. Hum. Percept. Perform. 26, 127–140 10.1037/0096-1523.26.1.127 10696609

[B143] Ratcliff R. Schmiedek F. McKoon G. (2008). A diffusion model explanation of the worst performance rule for reaction time and IQ. Intelligence 36, 10–17 10.1016/j.intell.2006.12.002 18584065 PMC2440712

[B144] Ratcliff R. Smith P. L. (2004). A comparison of sequential sampling models for two-choice reaction time. Psychol. Rev. 111, 333–367 10.1037/0033-295X.111.1.159 15065913 PMC1440925

[B145] Ratcliff R. Thapar A. McKoon G. (2001). The effects of aging on reaction time in a signal detection task. Psychol. Aging 16, 323–341 10.1037/0882-7974.16.2.323 11405319

[B146] Ratcliff R. Thapar A. McKoon G. (2006). Aging, practice, and perceptual tasks: a diffusion model analysis. Psychol. Aging 21, 353–371 10.1037/0882-7974.21.2.353 16768580 PMC2386252

[B147] Ratcliff R. Thapar A. McKoon G. (2007). Application of the diffusion model to two-choice tasks for adults 75–90 years old. Psychol. Aging 22, 56–66 10.1037/0882-7974.22.1.56 17385983 PMC2398682

[B148] Ratcliff R. Van Zandt T. McKoon G. (1999). Connectionist and diffusion models of reaction time. Psychol. Rev. 106, 261–300 10.1037/0033-295X.106.2.261 10378014

[B149] Rinkenauer G. Osman A. Ulrich R. Muller-Gethmann H. Mattes S. (2004). On the locus of speed-accuracy trade-off in reaction time: inferences from the lateralized readiness potential. J. Exp. Psychol. Gen. 133, 261–282 10.1037/0096-3445.133.2.261 15149253

[B150] Roberts S. (1981). Isolation of an internal clock. J. Exp. Psychol. Anim. Behav. Process. 7, 242–268 10.1037/0097-7403.7.3.242 7252428

[B151] Roitman J. D. Shadlen M. N. (2002). Response of neurons in the lateral intraparietal area during a combined visual discrimination reaction time task. J. Neurosci. 22, 9475–9489 12417672 10.1523/JNEUROSCI.22-21-09475.2002 PMC6758024

[B152] Roskies A. L. (2010). How does neuroscience affect our conception of volition? Annu. Rev. Neurosci. 33, 109–130 10.1146/annurev-neuro-060909-153151 20572769

[B153] Salthouse T. A. (1996). The processing-speed theory of adult age differences in cognition. Psychol. Rev. 103, 403–428 10.1037/0033-295X.103.3.403 8759042

[B154] Salzman C. Murasugi C. Britten K. Newsome W. (1992). Microstimulation in visual area MT: effects on direction discrimination performance. J. Neurosci. 12, 2331–2355 1607944 10.1523/JNEUROSCI.12-06-02331.1992 PMC6575906

[B155] Salzman C. D. Britten K. H. Newsome W. T. (1990). Cortical microstimulation influences perceptual judgements of motion direction. Nature 346, 174–177 10.1038/346174a0 2366872

[B156] Samejima K. Ueda Y. Doya K. Kimura M. (2005). Representation of action-specific reward values in the striatum. Science 310, 1337–1340 10.1126/science.1115270 16311337

[B157] Schall J. D. (2002). The neural selection and control of saccades by the frontal eye field. Philos. Trans. R. Soc. Lond. B Biol. Sci. 357, 1073–1082 10.1098/rstb.2002.1098 12217175 PMC1693021

[B158] Schall J. D. Thompson K. G. (1999). Neural selection and control of visually guided eye movements. Annu. Rev. Neurosci. 22, 241–259 10.1146/annurev.neuro.22.1.241 10202539

[B159] Schmiedek F. Oberauer K. Wilhelm O. Süss H.-M. Wittmann W. W. (2007). Individual differences in components of reaction time distributions and their relations to working memory and intelligence. J. Exp. Psychol. Gen. 136, 414–429 10.1037/0096-3445.136.3.414 17696691

[B160] Schouten J. F. Bekker J. A. M. (1967). Reaction time and accuracy. Acta Psychol. (Amst.) 27, 143–153 10.1016/0001-6918(67)90054-6 6062205

[B161] Shadlen M. N. Newsome W. T. (2001). Neural basis of a perceptual decision in the parietal cortex (area LIP) of the rhesus monkey. J. Neurophysiol. 86, 1916–1936 11600651 10.1152/jn.2001.86.4.1916

[B162] Simen P. (2012). Evidence accumulator or decision threshold – which cortical mechanism are we observing? Front. Psychol. 3:183 10.3389/fpsyg.2012.00183 22737136 PMC3380269

[B163] Simen P. Balci F. Desouza L. Cohen J. D. Holmes P. (2011). A model of interval timing by neural integration. J. Neurosci. 31, 9238–9253 10.1523/JNEUROSCI.3121-10.2011 21697374 PMC3142662

[B164] Simen P. Cohen J. D. Holmes P. (2006). Rapid decision threshold modulation by reward rate in a neural network. Neural Netw. 19, 1013–1026 10.1016/j.neunet.2006.05.038 16987636 PMC1808344

[B165] Simen P. Contreras D. Buck C. Hu P. Holmes P. Cohen J. D. (2009). Reward rate optimization in two-alternative decision making: empirical tests of theoretical predictions. J. Exp. Psychol. Hum. Percept. Perform. 35, 1865–1897 10.1037/a0016926 19968441 PMC2791916

[B166] Sirigu A. Daprati E. Ciancia S. Giraux P. Nighoghossian N. Posada A. Haggard P. (2004). Altered awareness of voluntary action after damage to the parietal cortex. Nature Neurosci. 7, 80–84 10.1038/nn1160 14647290

[B167] Smith P. L. (1995). Psychophysically principled models of visual simple reaction time. Psychol. Rev. 102, 567–593 10.1037/0033-295X.102.3.567

[B168] Smith P. L. (2010). From poisson shot noise to the integrated Ornstein–Uhlenbeck process: neurally principled models of information accumulation in decision-making and response time. J. Math. Psychol. 54, 266–283 10.1016/j.jmp.2009.06.007

[B169] Smith P. L. McKenzie C. R. L. (2011). Diffusive information accumulation by minimal recurrent neural models of decision making. Neural Comput. 23, 2000–2031 10.1162/NECO_a_00150 21521041

[B170] Smith P. L. Ratcliff R. (2004). Psychology and neurobiology of simple decisions. Trends Neurosci. 27, 161–168 10.1016/j.tins.2004.07.004 15036882

[B171] Smith Y. Bevan M. D. Shink E. Bolam J. P. (1998). Microcircuitry of the direct and indirect pathways of the basal ganglia. Neuroscience 86, 353–387 10.1016/S0306-4522(97)00608-8 9881853

[B172] Soon C. S. Brass M. Heinze H.-J. Haynes J.-D. (2008). Unconscious determinants of free decisions in the human brain. Nat. Neurosci. 11, 543–545 10.1038/nn.2112 18408715

[B173] Spaniol J. Madden D. J. Voss A. (2006). A diffusion model analysis of adult age differences in episodic and semantic long-term memory retrieval. J. Exp. Psychol. Learn Mem. Cogn. 32, 101–117 10.1037/0278-7393.32.1.101 16478344 PMC1894899

[B174] Starns J. J. Ratcliff R. (2010). The effects of aging on the speed-accuracy compromise: boundary optimality in the diffusion model. Psychol. Aging 25, 377–390 10.1037/a0018022 20545422 PMC2896207

[B175] Stone M. (1960). Models for choice-reaction time. Psychometrika 25, 251–260 10.1007/BF02289729

[B176] Swensson R. G. (1972). The elusive tradeoff: speed vs accuracy in visual discrimination tasks. Percept. Psychophys. 12, 16–32 10.3758/BF03212837

[B177] Thapar A. Ratcliff R. McKoon G. (2003). A diffusion model analysis of the effects of aging on letter discrimination. Psychol. Aging 18, 415–429 10.1037/0882-7974.18.3.415 14518805 PMC1360152

[B178] Townsend J. T. Ashby F. G. (1983). The Stochastic Modeling of Elementary Psychological Processes. Cambridge: Cambridge University Press

[B179] Tsetsos K. Gao J. McClelland J. L. Usher M. (2012). Using time-varying evidence to test models of decision dynamics: bounded diffusion vs. the leaky competing accumulator model. Front. Neurosci. 6:79 10.3389/fnins.2012.00079 22701399 PMC3372959

[B180] Tsetsos K. Usher M. McClelland J. L. (2011). Testing multi-alternative decision models with non-stationary evidence. Front. Neurosci. 5:63 10.3389/fnins.2011.00063 21603227 PMC3093747

[B181] Uhlenbeck G. Ornstein L. (1930). On the theory of the brownian motion. Phys. Rev. 36, 823–841 10.1103/PhysRev.36.823

[B182] Usher M. Elhalal A. McClelland J. L. (2008). “The neurodynamics of choice, value-based decisions, and preference reversal,” in The Probabilistic Mind: Prospects for Bayesian Cognitive Science, eds Chater N. Oaksford M. (Oxford: Oxford University Press), 277–300

[B183] Usher M. McClelland J. L. (2001). The time course of perceptual choice: the leaky, competing accumulator model. Psychol. Rev. 108, 550–592 10.1037/0033-295X.108.3.550 11488378

[B184] Usher M. McClelland J. L. (2004). Loss aversion and inhibition in dynamical models of multialternative choice. Psychol. Rev. 111, 757–769 10.1037/0033-295X.111.3.757 15250782

[B185] Van Essen D. C. Drury H. A. Dickson J. Harwell J. Hanlon D. Anderson C. H. (2001). An integrated software suite for surface-based analyses of cerebral cortex. J. Am. Med. Inform. Assoc. 8, 443–459 10.1136/jamia.2001.0080443 11522765 PMC131042

[B186] van Maanen L. Brown S. D. Eichele T. Wagenmakers E.-J. Ho T. Serences J. Forstmann B. U. (2011). Neural correlates of trial-to-trial fluctuations in response caution. J. Neurosci. 31, 17488–17495 10.1523/JNEUROSCI.2924-11.2011 22131410 PMC6623798

[B187] van Ravenzwaaij D. Oberauer K. (2009). How to use the diffusion model: parameter recovery of three methods: EZ, fast-dm, and DMAT. J. Math. Psychol. 53, 463–473 10.1016/j.jmp.2009.09.004

[B188] van Ravenzwaaij D. van der Maas H. L. J. Wagenmakers E.-J. (2012). Optimal decision making in neural inhibition models. Psychol. Rev. 119, 201–215 10.1037/a0026275 22103672

[B189] van Veen V. Krug M. K. Carter C. S. (2008). The neural and computational basis of controlled speed-accuracy tradeoff during task performance. J. Cogn. Neurosci. 20, 1952–1965 10.1162/jocn.2008.20146 18416686

[B190] Vickers D. (1970). Evidence for an accumulator model of psychophysical discrimination. Ergonomics 13, 37–58 10.1080/00140137008931117 5416868

[B191] Wagenmakers E.-J. Grasman R. P. P. P. Molenaar P. C. M. (2005). On the relation between the mean and the variance of a diffusion model response time distribution. J. Math. Psychol. 49, 195–204 10.1016/j.jmp.2005.02.003

[B192] Wagenmakers E.-J. Maas H. L. J. Grasman R. P. P. P. (2007). An EZ-diffusion model for response time and accuracy. Psychon. Bull. Rev. 14, 3–22 10.3758/BF03194105 17546727

[B193] Wagenmakers E.-J. Ratcliff R. Gomez P. McKoon G. (2008). A diffusion model account of criterion shifts in the lexical decision task. J. Mem. Lang. 58, 140–159 10.1016/j.jml.2007.04.006 19122740 PMC2330283

[B194] Wald A. (1947). Sequential Analysis. New York: Wiley

[B195] Wald A. Wolfowitz J. (1948). Optimum character of the sequential probability ratio test. Ann. Math. Stat. 19, 326–339 10.1214/aoms/1177730288

[B196] Wallsten T. S. Barton C. (1982). Processing probabilistic multidimensional information for decisions. J. Exp. Psychol. Learn. Mem. Cogn. 8, 361–384 10.1037/0278-7393.8.5.361

[B197] Wang X.-J. (2002). Probabilistic decision making by slow reverberation in cortical circuits. Neuron 36, 955–968 10.1016/S0896-6273(02)01092-9 12467598

[B198] Wenzlaff H. Bauer M. Maess B. Heekeren H. R. (2011). Neural characterization of the speed-accuracy tradeoff in a perceptual decision-making task. J. Neurosci. 31, 1254–1266 10.1523/JNEUROSCI.4000-10.2011 21273410 PMC6623618

[B199] Wickelgren W. A. (1977). Speed-accuracy tradeoff and information processing dynamics. Acta Psychol. (Amst.) 41, 67–85 10.1016/0001-6918(77)90012-9

[B200] Wiener N. (1923). Differential space. J. Math. Phys. 2, 131–174

[B201] Wong K.-F. Huk A. C. Shadlen M. N. Wang X.-J. (2007). Neural circuit dynamics underlying accumulation of time-varying evidence during perceptual decision making. Front. Comput. Neurosci. 1:6 10.3389/neuro.10.006.2007 18946528 PMC2525934

[B202] Wong K.-F. Wang X.-J. (2006). A recurrent network mechanism of time integration in perceptual decisions. J. Neurosci. 26, 1314–1328 10.1523/JNEUROSCI.0301-06.2006 16436619 PMC6674568

[B203] Yang T. Maunsell J. H. R. (2004). The effect of perceptual learning on neuronal responses in monkey visual area V4. J. Neurosci. 24, 1617–1626 10.1523/JNEUROSCI.4442-03.2004 14973244 PMC6730469

[B204] Yellott J. (1971). Correction for fast guessing and the speed-accuracy tradeoff in choice reaction time. J. Math. Psychol. 8, 159–199 10.1016/0022-2496(71)90011-3

[B205] Zeki S. (2007). The response properties of cells in the middle temporal area (Area MT) of owl monkey visual cortex. Proc. R. Soc. Lond. B Biol. Sci. 207, 239–248 10.1098/rspb.1980.0022 6102766

[B206] Zhang J. Bogacz R. (2010a). Bounded Ornstein–Uhlenbeck models for two-choice time controlled tasks. J. Math. Psychol. 54, 322–333 10.1016/j.jmp.2010.03.001 PMC2757788 19812713

[B207] Zhang J. Bogacz R. (2010b). Optimal decision making on the basis of evidence represented in spike trains. Neural Comput. 22, 1113–1148 10.1162/neco.2009.05-09-1025 20028228

[B208] Zhang J. Bogacz R. Holmes P. (2009). A comparison of bounded diffusion models for choice in time controlled tasks. J. Math. Psychol. 53, 231–241 10.1016/j.jmp.2009.03.001 19812713 PMC2757788

[B209] Zhang J. Hughes L. E. Rowe J. B. (2012). Selection and inhibition mechanisms for human voluntary action decisions. NeuroImage. 10.1016/j.neuroimage.2011.11.023 PMC3445813 22776456

[B210] Zhang J. Kourtzi Z. (2010). Learning-dependent plasticity with and without training in the human brain. Proc. Natl. Acad. Sci. U.S.A. 107, 13503–13508 10.1073/pnas.0910179107 20628009 PMC2922179

[B211] Zhang J. Meeson A. Welchman A. E. Kourtzi Z. (2010). Learning alters the tuning of functional magnetic resonance imaging patterns for visual forms. J. Neurosci. 30, 14127–14133 10.1523/JNEUROSCI.1039-10.2010 20962233 PMC6634776

[B212] Zhou X. Wong-Lin K. Philip H. (2009). Time-varying perturbations can distinguish among integrate-to-threshold models for perceptual decision making in reaction time tasks. Neural Comput. 21, 2336–2362 10.1162/neco.2009.12-07-671 19416080 PMC2784641

